# Self-Sorting in Diastereomeric Mixtures of Functionalized Dipeptides

**DOI:** 10.1021/acs.biomac.3c00246

**Published:** 2023-05-31

**Authors:** Qingwen Guan, Kate McAulay, Tian Xu, Sarah E. Rogers, Charlotte Edwards-Gayle, Ralf Schweins, Honggang Cui, Annela M. Seddon, Dave J. Adams

**Affiliations:** † School of Chemistry, University of Glasgow, Glasgow G12 8QQ, U.K.; ‡ Department of Chemical and Biomolecular Engineering, Whiting School of Engineering, Johns Hopkins University, Baltimore, Maryland 21218, United States; § ISIS Pulsed Neutron Source, Rutherford Appleton Laboratory, Didcot, OX11 0QX, U.K.; ∥ Diamond Light Source, Harwell Science and Innovation Campus, Didcot OX11 0QX, U.K.; ⊥ Large Scale Structures Group, Institut Laue-Langevin, 71 Avenue des Martyrs, CS 20156, F-38042 Grenoble,CEDEX 9, France; # School of Physics, HH Wills Physics Laboratory, University of Bristol, Tyndall Avenue, Bristol BS8 1TL, U.K.

## Abstract

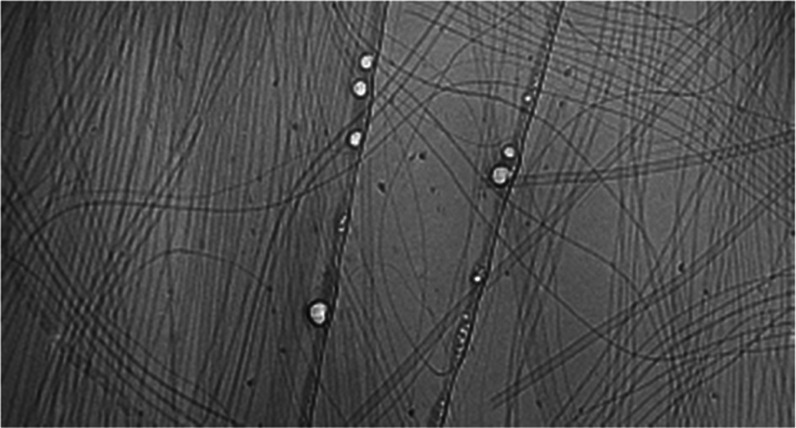

Self-sorting in functionalized dipeptide systems can be driven by the chirality of a single amino acid, both at a high pH in the micellar state and at a low pH in the gel state. The structures formed are affected to some degree by the relative concentrations of each component showing the complexity of such an approach. The structures underpinning the gel network are predefined by the micellar structures at a high pH. Here, we describe the systems prepared from two dipeptide-based gelators that differ only by the chirality of one of the amino acids. We provide firm evidence for self-sorting in the micellar and gel phases using small-angle neutron scattering and cryo-transmission electron microscopy (cryo-TEM), showing that complete self-sorting occurs across a range of relative concentrations.

## Introduction

**Figure 1:**
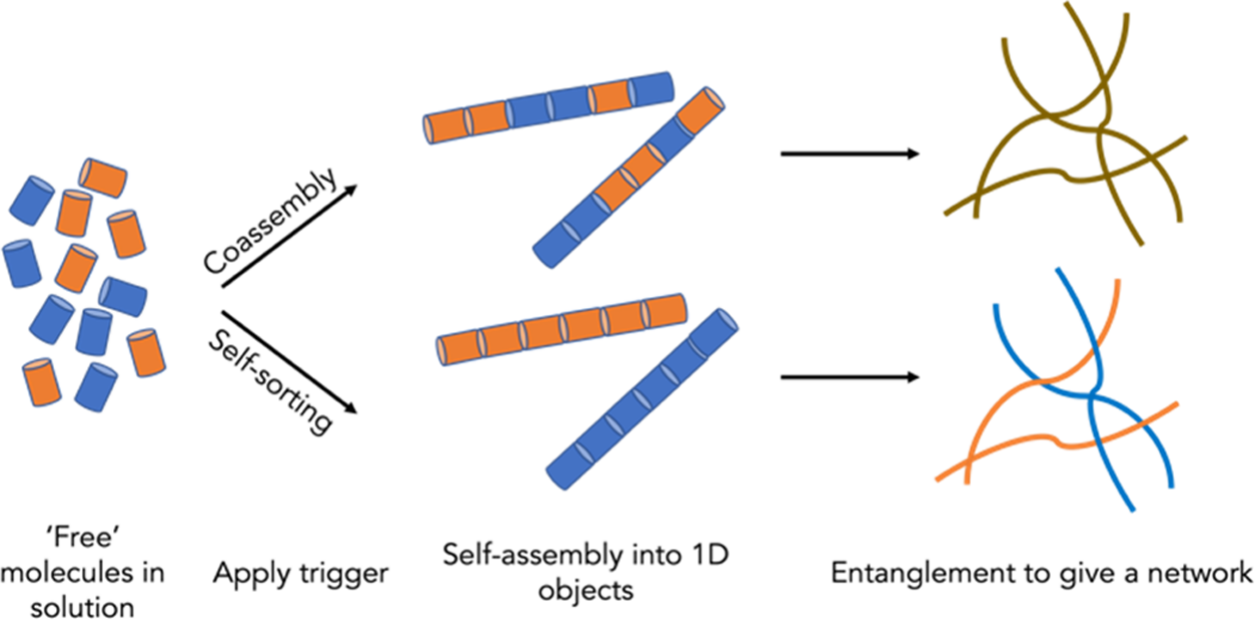
Cartoon showing how two gelators (shown as orange and blue) when mixed can form self-assembled fibers that (top) co-assemble and (bottom) self-sort.

Gels can be formed by the self-assembly of small molecules into fibers that entangle to give a network.^1−3^ In most cases, such gels are formed from a single species. However, interesting and useful materials can be prepared from multicomponent systems. When two molecules that can self-assemble alone are mixed to form a gel, multiple possibilities are available.^4,5^ Assuming a gel is still formed, first, the two molecules may mix in the self-assembled structures in either a specific or a random manner. Second, the two molecules may prefer to assemble independently, giving a self-sorted system (Figure 1). These possibilities refer to the primary self-assembled structures, with significant added complexity arising from how these structures can go on to further interact.^6^

Each of these systems has a potential use. For example, co-assembled systems can be used for cell work, whereby one component provides the matrix and the second component provides specific sites for cell adhesion or interaction.^7,8^ Self-sorted systems have been used to form optoelectronic systems^9−11^ and for advanced systems that can change properties on demand.^12^ Self-sorting can even be triggered to occur within cells.^13^

A key issue is the design of such systems.^4,5^ Limited methods exist that are known to drive a system toward a certain type of assembly. Specific mixing can be driven by mixing electron-rich and electron-poor gelators.^14^ Aside from this, there are examples where different types of systems are formed, but little in the way of design rules.

For self-sorted systems, many cases rely on the gelators having sufficiently different molecular structures,^15^ with the aim being that structural mismatch favors this assembly. In all of this, an important point is that most examples report a single set of conditions, which does not show that the design elements work across different concentrations and ratios. Further, the proof of what has been formed tends to rely on spectroscopy at low concentrations or microscopy, which can only at best provide a small snapshot of the structures formed. Some examples exist where gelators of different chirality exist. Here, again, there are limited design rules with some mixtures giving more robust gels than using a gelator of a single chirality,^16^ while other examples form mixtures where the different enantiomers or diastereomers disrupt the gelation.^17^ There are examples where co-assembly or self-sorting can be controlled by chirality.^18^

One method we have used to form self-sorted hydrogels relies on controlling the kinetics of self-assembly. Mixing two gelators with different apparent p*K*_a_ values for their terminal carboxylic acids combined with a slow decrease in pH is an effective method of forming self-sorted systems.^19^ There is however further complexity; at the initial high pH in water, micellar structures are formed, and we have found that in some cases, there is evidence of mixing in the micellar phases leading to some mixing in the gel state as the pH is decreased.^20^

Here, we describe the systems prepared from two dipeptide-based gelators that differ only by the chirality of one of the amino acids. We provide firm evidence for self-sorting in the micellar and the gel phase using small-angle neutron scattering and cryo-TEM, showing that complete self-sorting occurs across a range of relative concentrations.

## Experimental Section

### Materials

*N*-Boc-l-phenylalanine, l-phenylalanine methyl ester hydrochloride, d-phenylalanine methyl ester hydrochloride, and 2-(naphthalen-2-yloxy)acetic acid were obtained from Sigma-Aldrich; glucono-δ-lactone, 1,4-dioxane, and lithium hydroxide were purchased from Alfa Aesar; diethyl ether, *N*-methylmorpholine, sodium hydroxide, and hydrochloric acid were received from Honeywell; acetonitrile, chloroform, tetrahydrofuran, and sodium chloride were acquired from Fisher Scientific; hydrogen chloride (ca 4 mol/L in 1,4-dioxane) was obtained from Tokyo Chemical Industry; magnesium sulfate, dichloromethane, trifluoroacetic acid, and isobutyl chloroformate were purchased from VWR International, Fluorochem, and Thermo Scientific, respectively. All chemicals were used directly without further treatment. Deionized water was used throughout this research.

Full synthetic protocols and characterization data for the two gelators used here are described in the Supporting Information (Figures S1–S18).

### Stock Solutions

Stock solutions with a concentration of 10 mg/mL were prepared in a Falcon Tube by suspending 200 mg of 2NapFF in deionized water (15.97 mL) and adding 1 M sodium hydroxide solution (1 equiv, 4.03 mL) so that the molar ratio of sodium hydroxide and 2NapFF was kept as 1:1, and the total volume of the solution was 20 mL. Then, the solution was stirred at 1000 rpm overnight to ensure complete dissolution. Afterward, the pH of the solution was measured and adjusted to approximately 10.5 if needed with the addition of NaOH (2 M) or HCl (2 M) aqueous using a pipette with a full scale of 20 μL. Stock solutions with different volume ratios of (l,l)-, (l,d)-2NapFF were obtained by mixing the two stock solutions at different volumes and then stirring at 1000 rpm for 1 h. Subsequently, the pH of the mixtures was measured and adjusted to 10.5 if needed with the addition of NaOH (2 M) or HCl (2 M) aqueous using a pipette with a maximum scale of 20 μL.

### Gels

Gels were prepared in Sterilin vials (7 mL) by adding 2 mL of a stock solution prepared as above to glucono-δ-lactone (GdL) (16 mg/mL, 32 mg). The vials were gently rotated by hand to ensure the completed dissolution of GdL and left to stand overnight quiescently. Rheology data were collected 18 hours after the addition of GdL.

## Characterization

### pH Measurement

An FC200 pH probe (HANNA instruments) with a 6 mm × 10 mm conical tip was employed for all pH measurements. The precision of the pH measurements is stated as ±0.1.

### Optical Microscopy

Optical microscopic images (5× magnification) of solutions were obtained using a Nikon Eclipse LV100 microscope with a Nikon Plan ELWD 50×/0.60 lens attached to an Infinity2-1C camera. Images were taken with no polarizers (NP) or cross-polarizers (CP).

### UV–Vis Measurements

An Agilent Cary 60 UV–vis spectrophotometer (Agilent Technology, Selangor, Malaysia) was employed to record absorption spectra, using a 0.1 mm path length quartz demountable cuvette at 25 °C. For gels, 200 μL of pre-gelation solution containing GdL was introduced into the cuvette while in its liquid state. The cuvette was then sealed with Parafilm, and the sample was left to gel overnight before recording the spectrum.

### Turbidity Measurements

Using an Agilent Cary 60 UV–vis spectrophotometer (Agilent Technology, Selangor, Malaysia), the turbidity of all samples was measured at a wavelength of 600 nm with a 2 mm path length quartz cuvette at 25 °C. For gels, 1 mL of a pre-gelation solution containing GdL was placed into the cuvette while still in its liquid form. The cuvette was then sealed with Parafilm, and the sample was left to gel overnight before the spectrum was recorded.

### Rheology

An Anton Paar Physica MCR301 rheometer was used for rheological performance measurement.

### Viscosity Measurement

The viscosity was measured using the cone (CP50-1 18237) and plate system at 25 °C. The experiment gap distance between the cone and plate was fixed at 0.1 mm. Then, a 5 mL pipette and tip were used to transfer 1.5 mL of the solution onto the rheometer plate to minimize the shearing effect. The viscosity of each solution with the rotational shear rate was recorded, varying from 1 to 1000 s^–1^.

### Frequency and Strain Sweep

A vane (ST10-4V-8.8/97.5-SN42404) and cup system were used to directly measure the gap in a 7 mL plastic Sterilin vial containing 2 mL of gel sample was set to 1.8 mm during the entire test. Frequency sweep was measured from 1 to 100 rad s^–1^ at a fixed strain of 0.5%; strain sweep was measured from 0.1 to 1000% at a constant frequency of 10 rad s^–1^ to guarantee that 0.5% strain was located in the viscoelastic region required for the frequency sweep.

### Time Sweep

A cone (PP50/S 17154) and plate system were employed with the experiment gap at 0.8 mm. Then, 2 mL of the solution was added into a Sterilin vial containing preweighed 32 mg of GdL, and the vial was gently rotated to ensure that the GdL was completely dissolved. Subsequently, a 5 mL pipette tip was used to transfer the mixed solution onto the plate. To prevent the sample from evaporating, the edge of the cone was sealed with a small amount of mineral oil while avoiding the mineral oil from contacting the upper surface of the cone after the measurement proceeded for 15 min. Time sweep was measured at a constant frequency of 10 rad s^–1^ and a strain of 0.5% at 25 °C for 16 h.

### Circular Dichroism

CD spectra were collected using ChiraScan V× Spectrometer (Applied PhotoPhysics, UK). The solution was placed in 0.01 mm demountable High Precision Cells (Suprasil Quartz, Hellma Analytics, UK), and the corresponding spectrum between 300 and 180 nm was measured at 25 °C. The data spacing was 1 nm, the bandwidth was set to 1 nm, the scanning speed was 120 nm/min, and repeats were ticked as 3.

### Cryo-Transmission Electron Microscopy (cryo-TEM)

Cryogenic TEM imaging was performed either using FEI Tecnai 12 TWIN transmission electron microscope operated at 100 kV or using FEI Talos 200SC FEG that was operated at 200 kV. In general, a drop of the studied sample solution, approximately 7 μL, was placed on a holey carbon film supported on a TEM copper grid (Electron Microscopy Services, Hatfield, Pennsylvania). Prior to TEM sample preparation, all of the TEM grids used for cryo-TEM imaging were treated with plasma air to render the lacey carbon film hydrophilic. A vitrified thin film of the sample solution, typically less than 200 nm, was produced using the Vitrobot with a controlled humidity chamber (FEI). The vitrified samples were then transferred to a cryo-holder and cryo-transfer stage, cooled by liquid nitrogen. The cryo-holder temperature was maintained below −170 °C during the imaging process to prevent the sublimation of vitreous water. All images were recorded by an EMSIS Megaview G III wide-angle CCD camera or Thermo Scientific Ceta (CMOS) camera.

### Small-Angle Neutron Scattering

SANS measurements of the gelator solutions and gel samples were performed at either ISIS or the ILL. At ISIS, measurements were carried out using the SANS2D time-of-flight diffractometer (STFC ISIS Pulsed Neutron Source, Oxfordshire, UK). A simultaneous *Q* range of 0.005–1.0 Å^–1^ was achieved using an incident wavelength (λ) range of 1.75–16.5 Å and employing two 1 m^2^ detectors. The small-angle detector was positioned 4 m from the sample and offset vertically 80 mm and sideways 100 mm. The wide-angle detector was positioned 2.4 m from the sample, offset sideways by 980 mm, and rotated to face the sample. The incident neutron beam was collimated to 8 mm diameter. Samples were housed in 2 mm pathlength quartz cuvettes and measured for 60 min each. The “raw” scattering data were normalized to the incident neutron wavelength distribution, corrected for the linearity and efficiency of the detector response, and the measured neutron transmission (i.e., absorbance) using the Mantid framework.^21,22^ They were then placed on an absolute scale by comparison with the expected scattering from a partially deuterated polystyrene blend of known composition and molecular weights following established procedures. The background scattering from a quartz cell containing D2O was then subtracted.

Measurements using the small-angle neutron scattering instrument D11 at the Institut Laue–Langevin (ILL; Grenoble, France) were performed using a neutron wavelength of 10 Å and three sample-to-detector distances of 39, 8, and 1.2 m (with respective collimation distances of 40.5, 8, and 5.5 m). An MWPC 3He detector consisting of 128 × 128 pixels of 7.5 × 7.5 mm^2^ size was used. The employed neutron beam was 13 mm in diameter. The thermostated rack was kept at 20 °C. Data reduction was performed using the facility-provided software LAMP. Data have been put on absolute scale by measuring the secondary calibration standard H_2_O (1 mm thickness), cross-calibrated against h/d polymer blends, with the known differential scattering cross section of 1.245 1/cm for 10 Å.

### Small-Angle X-ray Scattering

SAXS measurements were performed at Diamond Light Source (Oxfordshire, UK) on the B21 beamline.^23^ Samples were loaded into 1.5 mm diameter glass capillaries using a 1 mL syringe and a 19G needle immediately after GdL addition, allowing gelation in the capillary. The capillaries were sealed with parafilm, loaded into a three-dimensional (3D) printed cell, and then into the instrument via the multipurpose sample (MPS) cell.^24^ 20 × 1 s frames were collected on the samples. The X-ray beam possessed a wavelength of 0.9537 Å and an energy of 13 keV. An EigerX 4M (Dectris) detector was used at a sample-to-detector distance of 3712.7 mm, resulting in a *Q* range of 0.0026–0.34 Å^–1^. The data were processed in Dawn Science (version 2.25, https://dawnsci.org/). The scattering from deionized water in a glass capillary was used as the background. The two-dimensional (2D) images were azimuthally integrated to produce the 1D *I* vs *Q* plots.

## Results and Discussion

**Figure 2:**
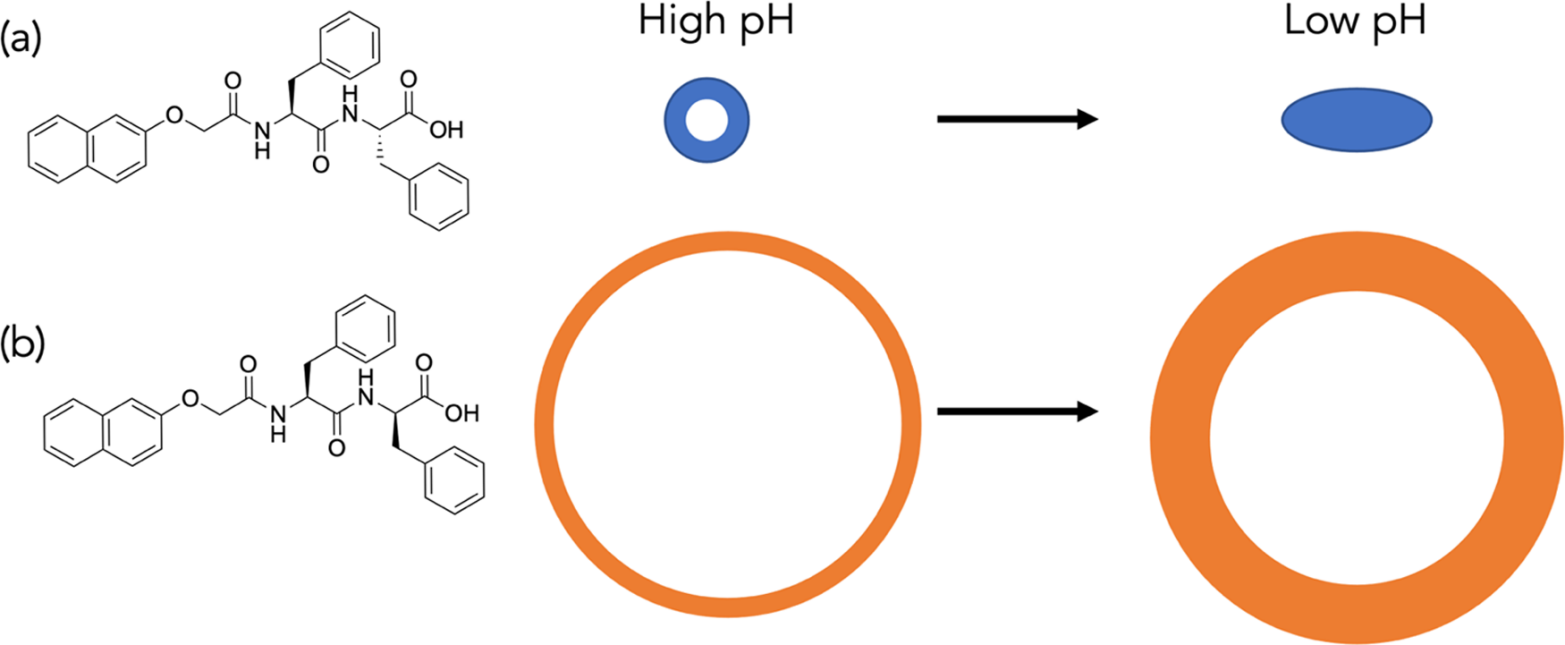
Chemical structures of (a) (l,l)-2NapFF and (b) (l,d)-2NapFF with to-scale cartoon of cross-sectional view of the nanotubes formed by each and the transition in structure on gelation. In the micellar state, (l,l)-2NapFF forms nanotubes with a core radius of 1.7 nm and a wall thickness of 1.9 nm; (l,d)-2NapFF forms nanotubes with a core radius of 13.4 nm and a wall thickness of 1.4 nm. On gelation, for (l,l)-2NapFF, the core collapses and lateral aggregation occurs, leading to the apparent formation of elliptical cylinders, whereas for (l,d)-2NapFF, large nanotubes persist as the pH decreases.

**Figure 3:**
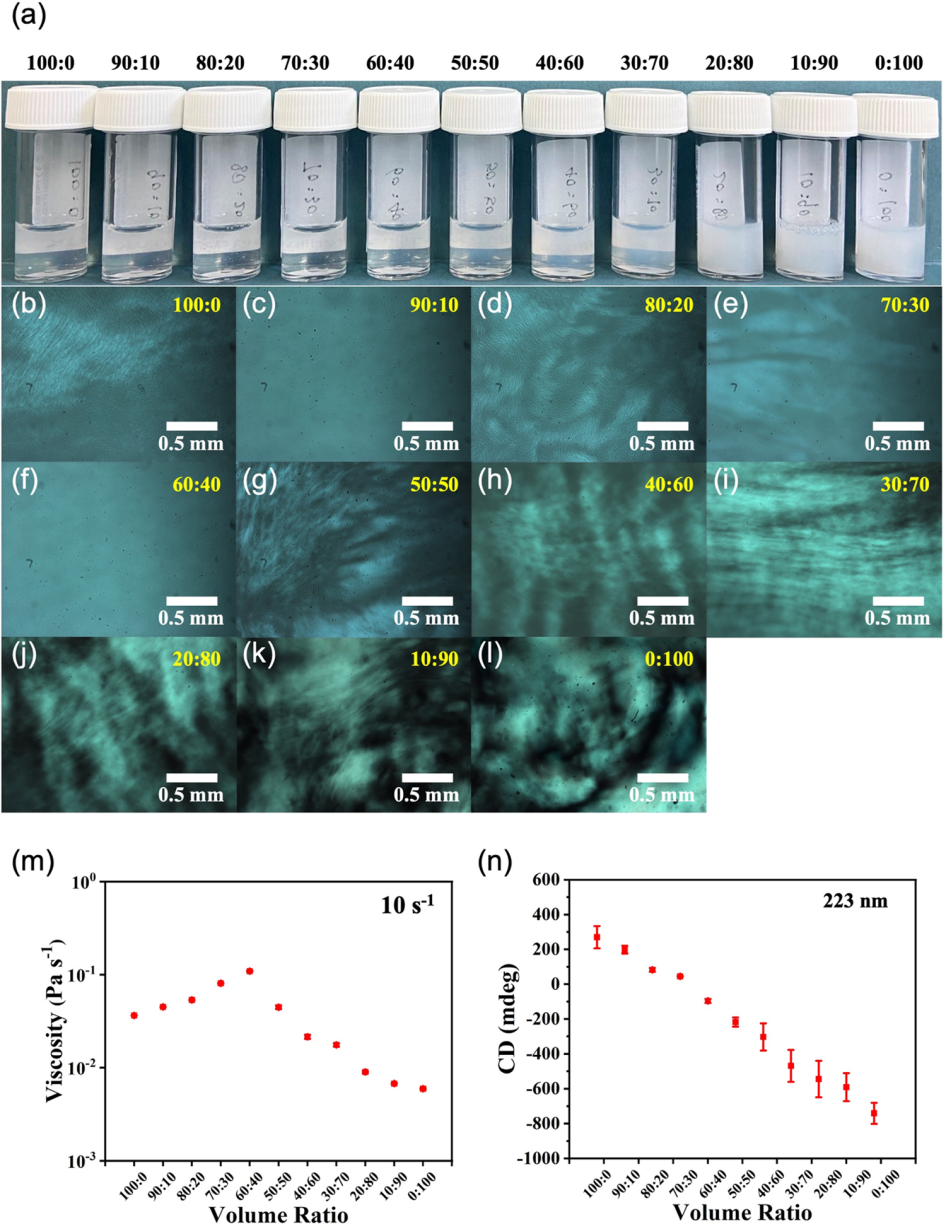
(a) Photographs of solutions of (l,l)-2NapFF and (l,d)-2NapFF are different ratios at an overall concentration of 10 mg/mL; (b–l) cross-polarized optical microscopy images of the solutions shown in (a) with ratios of (l,l)-2NapFF and (l,d)-2NapFF shown on each image in red text; (m) viscosity data at 10 s^–1^ and (n) CD data at 223 nm for solutions with various volume ratios of (l,l)- and (l,d)-2NapFF.

2NapFF is a robust gelator, forming micellar structures at a high pH and gels at a low pH.^25,26^ Where both phenylalanines are the l-enantiomer ((l,l)-2NapFF; Figure 2a), the small-angle neutron scattering (SANS) data show that at a high pH, nanotubes are formed with a core radius of 1.7 nm and a wall thickness of 1.9 nm.^25^ This is corroborated by cryo-TEM.^25^ Gels are formed when the pH is decreased, where the core of the nanotubes collapse and lateral aggregation of the resulting cylindrical structures occurs.^26^ We have also shown that the (l,d)-2NapFF diastereomer (Figure 2b) self-assembles into large, thin-walled rigid nanotubes at a high pH with a core radius of 13.4 nm and a wall thickness of 1.4 nm as shown by SANS and again corroborated by cryo-TEM.^25^ When the pH is decreased, gels form where these structures persist. Considering the very different structures present in the single-component systems at both high and low pH values, we hypothesized that mixing (l,l)-2NapFF and (l,d)-2NapFF might result in self-sorted systems based on the morphology of self-assembled structure despite the similarity in molecular structure.

(l,l)-2NapFF and (l,d)-2NapFF were prepared as described previously.^25^ Solutions of each were prepared at a concentration of 10 mg/mL at a pH of 10.5. At this concentration, both (l,l)-2NapFF and (l,d)-2NapFF form liquid crystal phases as shown by polarized microscopy, with (l,d)-2NapFF forming a more turbid solution by eye (Figure 3a). The solutions of (l,l)-2NapFF and (l,d)-2NapFF at a high pH were mixed to give solutions at several different ratios with a constant overall concentration of 2NapFF of 10 mg/mL. Visually, the turbidity decreased as the composition of the (l,l)-2NapFF increased.

Polarized light microscopy images (Figures 3b–l and S19) showed significant birefringence for ratios of (l,l)-2NapFF:(l,d)-2NapFF of 50:50 to 0:100. Birefringent domains are also observed in solutions for the other ratios excluded 90:10 and 60:40. The viscosity varied across the series, with the mixture at 60:40 (l,l-)2NapFF:(l,d)-2NapFF being the most viscous (Figures 3m and S20). The turbidity of the samples increased as the amount of (l,d)-2NapFF in the mixtures was increased (Figure S21).

Circular dichroism spectra for solutions of (l,l)-2NapFF and (l,d)-2NapFF showed different signals. The CD spectra of (l,d)-2NapFF were much weaker than the (l,l)-2NapFF. In the mixtures, the CD signals became less intense as the ratio of (l,d)-2NapFF increased (Figures S22, S23, and Tables S1–S3). In all cases, the spectra were dominated by the phenylalanine and naphthalene rings with signals between 200 and 230 nm, with a maximum wavelength of 225 nm and a wavelength range of 240–290 nm.^27,28^ The signal intensity at 223 nm declined almost linearly with the increasing addition of (l,d)-2NapFF (Figure 3n).

**Figure 4:**
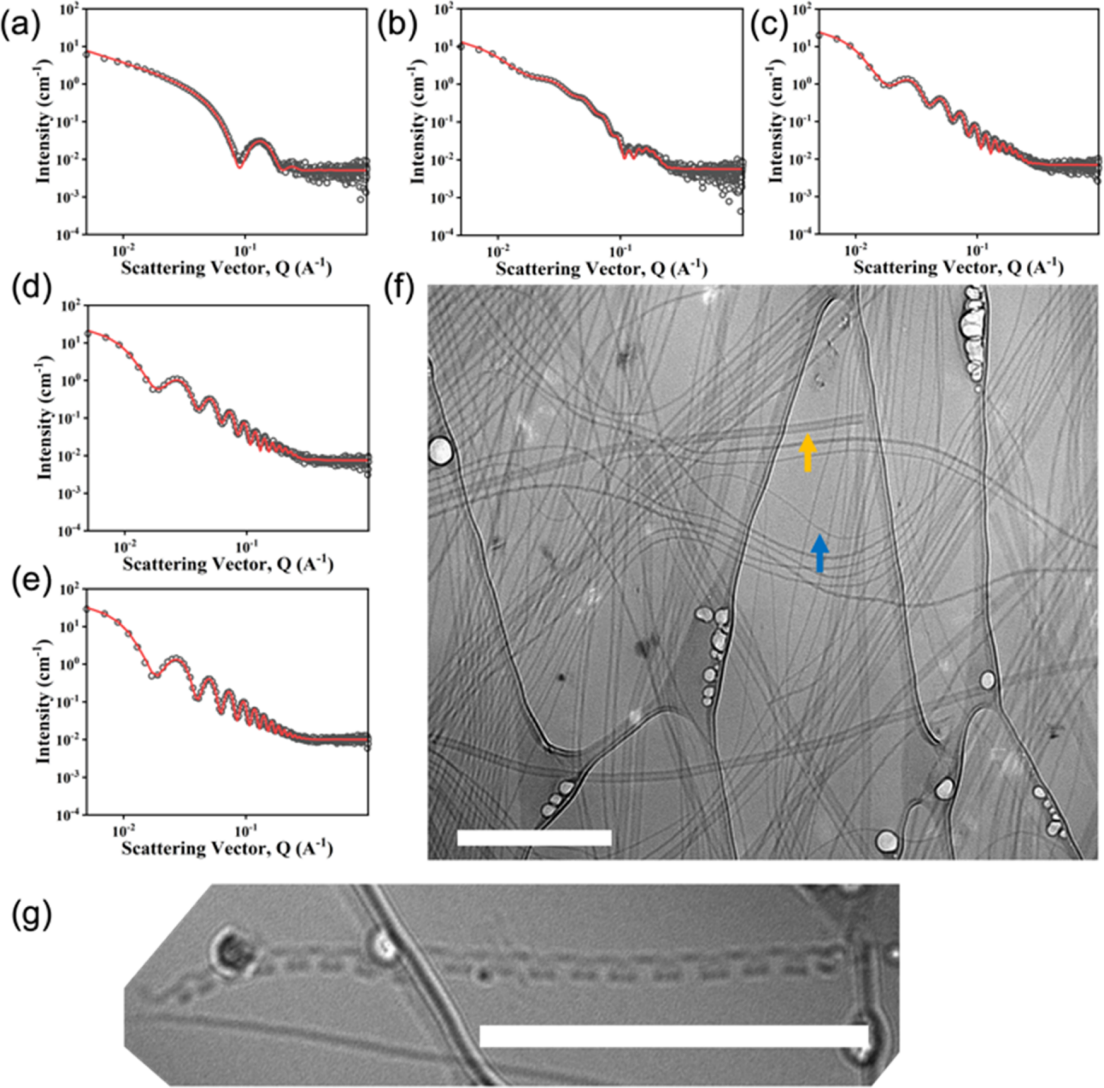
SANS data (black circles) with fits to a two-cylinder model (full fit parameters in Table S1) for ratios of (l,l)-2NapFF:(l,d)-2NapFF of (a) 100:0; (b) 70:30; (c) 50:50; (d) 30:70; and (e) 0:100. (f) Cryo-TEM image of the 30:70 mixture showing the coexistence of large nanotubes (one highlighted in orange) and thinner structures (one highlighted by a blue arrow). The scale bar for (f) represents 500 nm. (g) Zoom-in view of a cryo-TEM image showing the formation of a nanotube. The scale bar is 500 nm.

To understand these systems in more detail, we collected SANS data for mixtures of the (l,l)-2NapFF and (l,d)-2NapFF (Figure S24). The data for the single-component systems fitted well to hollow cylinder models as previously described and summarized above (Table S4).^25^ The data for the mixtures were successfully fit to a combination of two hollow cylinders (Table S4). One of these has parameters that are identical to those of the (l,d)-2NapFF alone across this range of composition and concentration with a core of 13.4 nm and a wall thickness of 1.4 nm. The parameters for the second hollow cylinder depend on the exact composition and concentration (Figure 4). It therefore appears that the (l,d)-2NapFF robustly forms the same structures (Figure S25) while the structures formed by (l,l)-2NapFF are affected by composition and concentration. We can rule out our changes in concentration leading to these changes by comparing to data for the (l,l)-2NapFF alone (see Figure S26). At lower concentrations, for example, 3 mg/mL, the SANS data fit best to a flexible cylinder model with a radius of around 3.1 nm. Hence, in the presence of the second component, the micellar structures at low concentrations of (l,l)-2NapFF are different from those formed by (l,l)-2NapFF alone, again exemplifying the complexity of these systems. Cryo-TEM images corroborate the SANS data. Two populations of the structure are found in all mixtures examined (Figure S27 and Table S5). Hence, at a high pH, we have a self-sorted micellar system with two co-existing populations. We highlight that this is not always the case; we have recently shown that two structurally dissimilar functionalized dipeptides form mixed micelles at a high pH.^29^ We also note that due to operational issues, the cryo-TEM data were collected around 1 year after sample formation, implying that there is no kinetic trapping occurring.

An interesting case was observed for one case where we were able to observe the formation of nanotubes from (l,d)-2NapFF. In one image (Figure 4g), there is clear evidence that these nanotubes are formed by the wrapping up of a tape-like structure, presumably formed from a bilayer as has been observed for other systems.^30,31^

It is worth noting that we have worked with other examples where co-assembly occurs^20,29^—in these cases, the molecules are very different, but the micellar structures are a wormlike micelle and a spherical micelle. Since very different molecules can co-assemble at a high pH^20,29^ and (in some cases also at a low pH^20^), the self-sorting here does not seem to be driven simply by the small differences in molecular structure, but rather by the fact that both of these structures form robust nanotubes at a high pH. However, further work is needed with other systems to understand this. It may be that mixing preformed micellar solutions as is done here also favors self-sorting and that it may be possible to drive more toward co-assembly by direct dissolution of a mixture of the two solids.^32^

**Figure 5:**
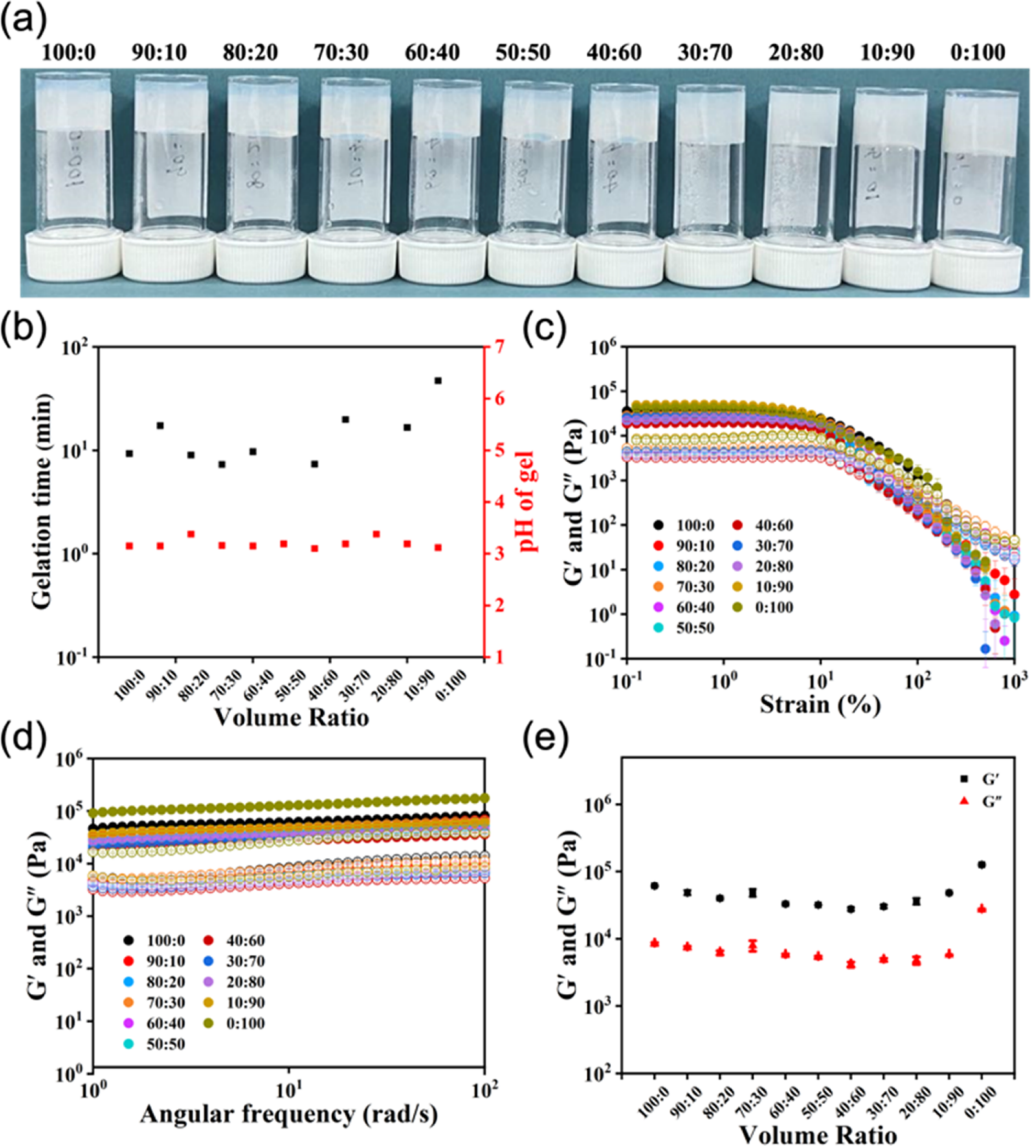
(a) Photographs, (b) plots of gelation time and pH of gel versus volume ratio, (c) strain, (d) frequency sweep, and (e) modulus at 10 rad s^–1^ of gels formed by solutions with various volume ratios of (l,l)-, and (l,d)-2NapFF. For (c) and (d), full symbols represent *G*′ and open symbols represent *G*″. In all cases for (b)–(e), the data points represent the average value of the experimental data of the three samples, and the error bar represents their standard deviation.

Gels were then prepared from the solutions containing different ratios of (l,l)-, and (l,d)-2NapFF by lowering the pH slowly and controllably using the hydrolysis of GdL to gluconic acid to achieve reproducible and homogeneous kinetics of pH change and gelation.^33,34^ Vial inversion showed that all of the ratios formed self-supporting materials (Figure 5a). Consistent with our previous report, (l,l)-2NapFF started to gel earlier than (l,d)-2NapFF (Figure 5b) on the basis of the higher p*K*_a_ of the terminal carboxylic acid.^25^ Following the gelation by rheology with time, both the storage modulus (*G*′) and loss modulus (*G*″) gradually increased as the assembly progressed and became essentially constant after about 5 h (Figure S28 and Table S6) except for ratios of 10:90 and 0:100, which took around 10 and 15 h, respectively. The gradual conversion of the viscous solutions to a gel was associated with the decrease in pH, with the resulting gels having a final pH of 3.1–3.4 (Table S5). A final gel was confirmed by the linear viscoelastic region in the strain sweep of the final gels (Figure 5c), and both *G*′ and *G*″ being frequency-independent (Figure 5d). The final values of *G*′ and *G*″ varied slightly with the ratio of (l,l)-2NapFF and (l,d)-2NapFF. The turbidity of the gels increased as the concentration of (l,d)-2NapFF in the mixture increased (Figure S29).

To understand the gel phase, we again used SANS to probe the underlying structures (Figure S30 and Table S7). The data for the (l,l)-2NapFF alone agree with our previous data,^26^ showing that the network is formed by structures that best fit to a flexible elliptical cylinder model. For (l,l)-2NapFF, as the pH decreases, the core first collapses, followed by lateral association of the resulting cylinders.^26^ For the (l,d)-2NapFF, the structures best fit to a hollow cylinder model,^25^ showing that the micellar structures template the structures in the gel state. The SANS data for the mixtures rich in (l,d)-2NapFF (50:50 and higher) can be fit to a mixture of a hollow cylinder and flexible elliptical cylinder models, with the parameters for the hollow cylinder being close to that for the pure (l,d)-2NapFF. At lower concentrations, the data best fit to a flexible elliptical cylinder model alone. We interpret this as there being self-sorting in these systems, but the scattering being dominated by the l,l-2NapFF as this becomes the highest concentration species.

## Conclusions

Previous work examining multicomponent systems similar to those described here in water tends to focus on the gel state, with little if any discussion of the importance (or not) of any structures present prior to gelation.^7,35−39^ There seems to often be an assumption that there is complete dissolution prior to gelation, which we and others have shown is often not the case for such hydrophobic molecules that are charged at high pH.^32,40^ This is an interesting point—in most organogel systems, gels are formed by heating to dissolve the gelator and then cooling to form the gels. Similarly, for gelators such as those used in this work, gelation can often also be achieved by dissolution in a good solvent such as DMSO followed by the addition of water. Indeed, mixed assemblies have been prepared by such methods.^41−43^ In this scenario, there ought to be molecular dissolution prior to water addition and gelation.

Here, this pre-formation of a micellar dispersion at a high pH prior to gelation at a low pH provides different possibilities in terms of self-sorting and co-assembly (and scenarios in between) compared to direct dissolution. We show that the structures formed at a high pH can persist into the gel state and self-sorting can occur on the basis of the chirality of one of the amino acids. Chirality has been examined previously in low-molecular-weight gelling systems^44,45^ but generally as single components where the gelling efficiencies of different enantiomers and diastereomers have been compared.^46−48^ For example, Xu’s group has compared the l,l and d,d- analogues of naphthalene dipeptides from the perspective of in vivo hydrolysis.^49^ Here, we have shown that self-sorting can be driven by the chirality of a single amino acid in these functionalized dipeptide systems, with two distinct micellar structures being formed at a high pH. The structures formed are affected to some degree by the relative concentrations of each component showing the complexity of such an approach. This aspect of relative concentration is rarely discussed as a possible means of tuning the system in multicomponent systems. On lowering the pH, a gel is formed in all cases, with self-sorting again occurring. Hence, the structures underpinning the gel network are predefined by the micellar structures at a high pH, and differences here can be driven by a change in the chirality of a single amino acid. This work shows both the power and complexity of these systems.

## Supporting Information Available

Full synthesis and characterization details, further SAXS data, CD data, TEM images, and rheology data (DOCX)
