# Supplementary material for: Self-Sorting in Diastereomeric Mixtures of Functionalized Dipeptides
Source: Biomacromolecules. 2023 May 31;24(6):2847–55. doi: 10.1021/acs.biomac.3c00246 (PMC10265659; doi:10.1021/acs.biomac.3c00246)
Supplement: Supplementary file 1 — bm3c00246_si_001.pdf [file bm3c00246_si_001.pdf]

Self-sorting in Diastereomeric Mixtures of Functionalised Dipeptides

Qingwen Guan,[a] Kate McAulay,[a] Tian Xu,[b] Sarah E. Rogers,[c] Charlotte Edwards-Gayle,[d] Ralf Schweins,[e] Honggang Cui,[b] Annela M. Seddon[f] and Dave J. Adams[a,]*

[a] *School of Chemistry, University of Glasgow, Glasgow, G12 8QQ, UK. Email: dave.adams@glasgow.ac.uk*

[b] *Department of Chemical and Biomolecular Engineering, Whiting School of Engineering, Johns Hopkins University, Baltimore, Maryland 21218, United States.*

[c] *ISIS Pulsed Neutron Source, Rutherford Appleton Laboratory, Didcot OX11 0QX, U.K.*

[d] *Diamond Light Source, Harwell Science and Innovation Campus, OX11 0QX, UK*

[e] *Large Scale Structures Group, Institut Laue-Langevin, 71 Avenue des Martyrs, CS 20156, F-38042 Grenoble, CEDEX 9, France*

[f] *School of Physics, HH Wills Physics Laboratory, University of Bristol, Tyndall Avenue, Bristol, BS8 1TL, UK*

Supporting Information

Synthesis and Characterisation of Gelators

Synthesis of (L,L)-2NapFF

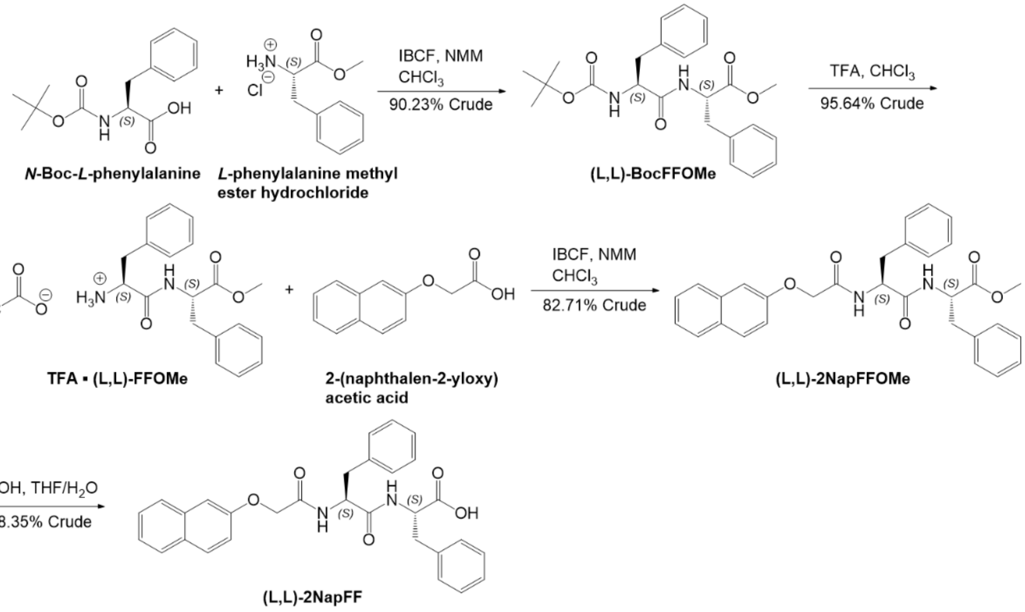

Figure S1. Synthesis route of (L,L)-2NapFF.

<u>Methyl (2*S*)-2-[(2*S*)-2-{[(*tert*-butoxy)carbonyl]amino}-3-phenylpropanamido]-3-phenylpropanoate</u>

To a solution of *N*-Boc-L-phenylalanine (2.65 g, 10 mmol) in chloroform (30 mL) was added *N*-methylmorpholine (2 eq., 2.20 mL) and isobutylchloroformate (1.1 eq., 1.43 mL), and the mixture was stirred on ice for 10 min. Meanwhile, to a suspension of L-phenylalanine methyl ester hydrochloride (2.92 g, 10 mmol) in chloroform (30 mL) was added *N*-methylmorpholine (2 eq., 2.20 mL) and the mixture was stirred at room temperature for 10 minutes. Subsequently, the latter mixture was added to the former and kept stirring overnight on ice. The resulting clear solution was diluted with chloroform and washed in turn with 100 mL of 1M hydrochloric acid, water, and brine (saturated), before being dried (magnesium sulfate), and the solvent removed under vacuum. (L,L)-BocFFOMe (3.85 g, 90%) was obtained as a white powder.

δ_H (400 MHz, DMSO-d_6, 25 °C) 8.38 (0.2H, d, *J* 6.68, Rot-1 N<u>H</u>CH*CO_2Me), 8.31 (0.8H, d, *J* 7.59, Rot-2 N<u>H</u>CH*CO_2Me), 7.30-7.16 (10.2H, m, <u>H</u>$_{Ar}$), 6.83 (0.8H, d, *J* 8.76, Rot-2 N<u>H</u>Boc), 6.39 (0.2H, d, *J* 7.89, Rot-1 N<u>H</u>Boc), 4.51 (1H, dd, *J* 13.88, 7.92, C<u>H</u>*NHBoc), 4.20 (0.8H, td, *J* 10.06, 4.23,

Rot-2 C$\underline{H}^*$CO$_2$Me), 4.08-4.02 (0.2H, Rot-1 C$\underline{H}^*$CO$_2$Me), 3.58 (3H, s, OC$\underline{H}_3$), 3.32 (2.9H, br s, $\underline{H}_2$O), 3.08-2.94 (2.1H, m, PhC$_a\underline{H}_2$), 2.89 (1H, dd, *J* 13.76, 4.12, PhC$_b\underline{H}_a$H$_b$), 2.67 (1H, dd, *J* 13.71, 10.49, PhC$_b$H$_a\underline{H}_b$), 2.50 (1.4H, quintet, residual DMSO-d$_5$), 1.28 (7.6H, s, Rot-2 C(C$\underline{H}_3$)$_3$), 1.15 (1.5H, s, Rot-1 C(C$\underline{H}_3$)$_3$). δ_C (100 MHz, DMSO-*d*$_6$, 25 °C) 171.87, 171.83, and 155.12 ($\underline{C}$=O), 138.03, 137.02, 129.18, 129.14, 128.28, 128.00, 126.60 and 126.18 ($\underline{C}_{Ar}$), 78.05 ($\underline{C}$(CH$_3$)$_3$), 55.53 ($\underline{C}$H*NHBoc), 53.53 ($\underline{C}$H*CO$_2$Me), 51.86 (CO$_2\underline{C}$H$_3$), 39.52 (septet, DMSO-d$_6$), 37.45 (Ph$\underline{C}_b$H$_2$), 36.74 (Ph$\underline{C}_a$H$_2$), 28.13 (Rot-2 C($\underline{C}$H$_3$)$_3$), 27.76 (Rot-1 C($\underline{C}$H$_3$)$_3$).

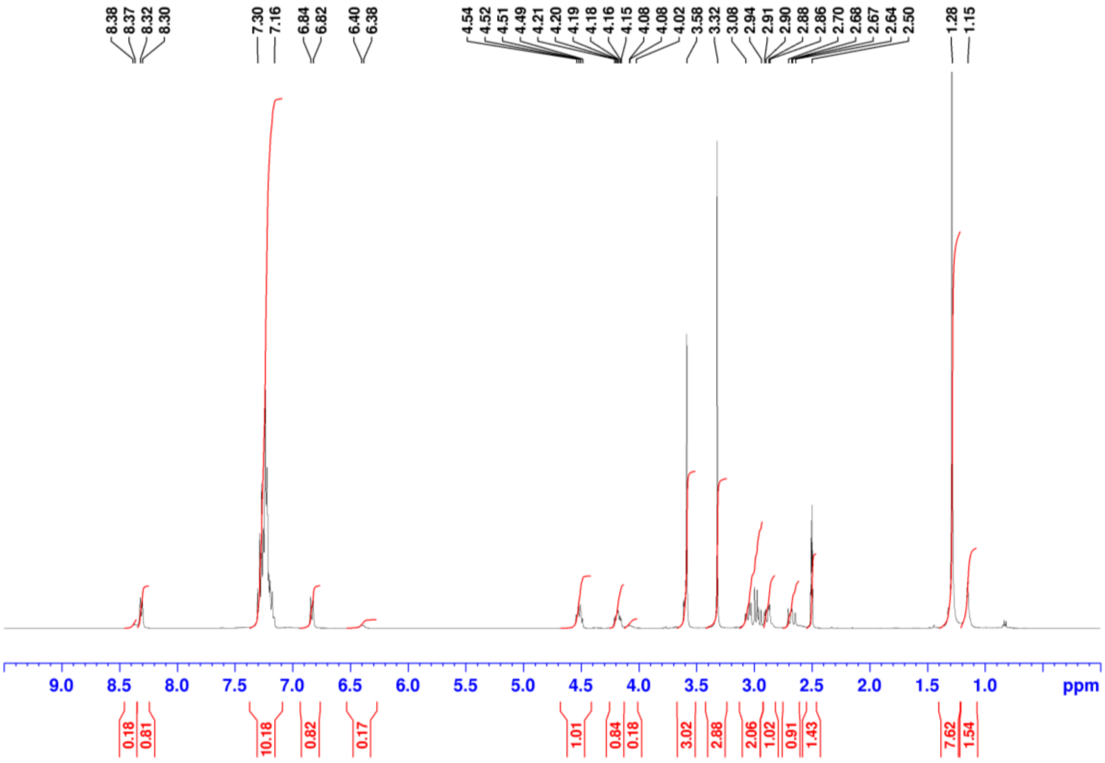

Figure S2. ^{1}H NMR spectrum of (L,L)-BocFFOMe in d$_6$-DMSO at 25 °C.

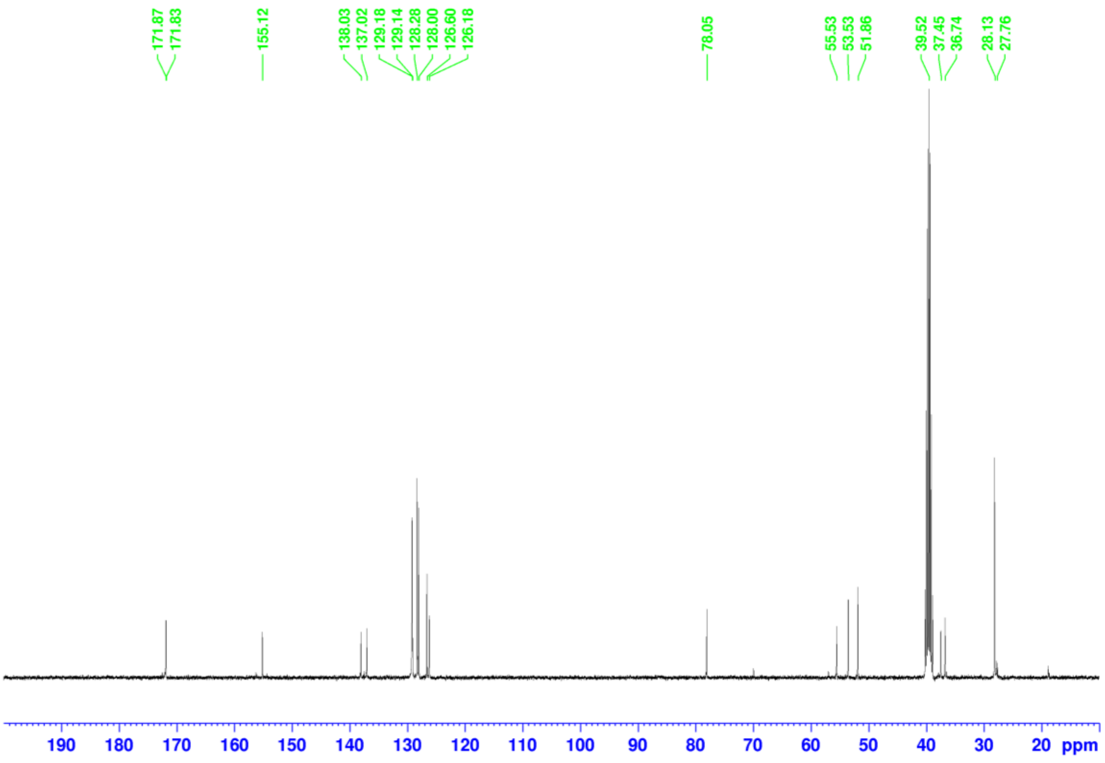

Figure S3. ^{13}C NMR spectrum of (L,L)-BocFFOMe in d$_6$-DMSO at 25 °C.

Methyl

(2*S*)-2-[(2*S*)-2-azaniumyl-3-phenylpropanamido]-3-phenylpropanoate trifluoroacetate salt

To a solution of (L,L)-BocFFOMe (3.85 g, 9.02 mmol) in chloroform (10 mL) was added trifluoroacetic acid (5 mL) and the mixture was stirred at room temperature overnight. The mixture was subsequently concentrated under reduced pressure to remove most of the excess TFA and the resulting viscous oil was then dissolved in ~30 mL of chloroform, transferred to ~200 mL of diethyl ether and stirred for 2 h to allow complete precipitation. The white solid was collected by filtration. The solid was washed with a small amount of diethyl ether (~10 mL) and dried under vacuum. TFA·(L,L)-FFOMe (3.81 g, 96%)) was collected as a white powder.

δ_H (400 MHz, DMSO-d$_6$, 25 °C) 9.07 (1H, d, *J* 7.48, N$\underline{H}$), 8.23 (2.8H, br s, N$\underline{H}_3^+$), 7.34-7.22 (9.8H, m, $\underline{H}_{Ar}$), 4.56 (1H, dd, *J* 14.15, 7.44, C$\underline{H}^*$NH$_3^+$), 4.09-4.06 (1H, m, C$\underline{H}^*$CO$_2$Me), 3.60 (3H, s, OC$\underline{H}_3$), 3.40 (5.7H, br s, $\underline{H}_2$O), 3.14-3.04 (2.0H, m, PhC$_a\underline{H}_2$), 3.00-2.93 (1.9H, m, PhC$_b\underline{H}_2$), 2.50 (0.7H, quintet, residual DMSO-d$_5$). δ_C (100 MHz, DMSO-d$_6$, 25 °C) 171.21 and 168.31 (CH-$\underline{C}$=O), 158.26 (q, *J* 31.01, CF$_3$-$\underline{C}$=O), 136.76, 134.77, 129.61, 129.12, 128.55, 128.45, 127.20, and 126.80 ($\underline{C}_{Ar}$),

117.80 (d, J 300.36, <u>C</u>F_3), 53.92 (<u>C</u>H^*CO_2Me), 53.18 (O<u>C</u>H_3), 52.07 (<u>C</u>$H^*NH_3^+$), 39.52 (septet, DMSO-d_6), 36.92 (Ph<u>C</u>$_aH_2$), 36.70 (Ph<u>C</u>$_bH_2$).

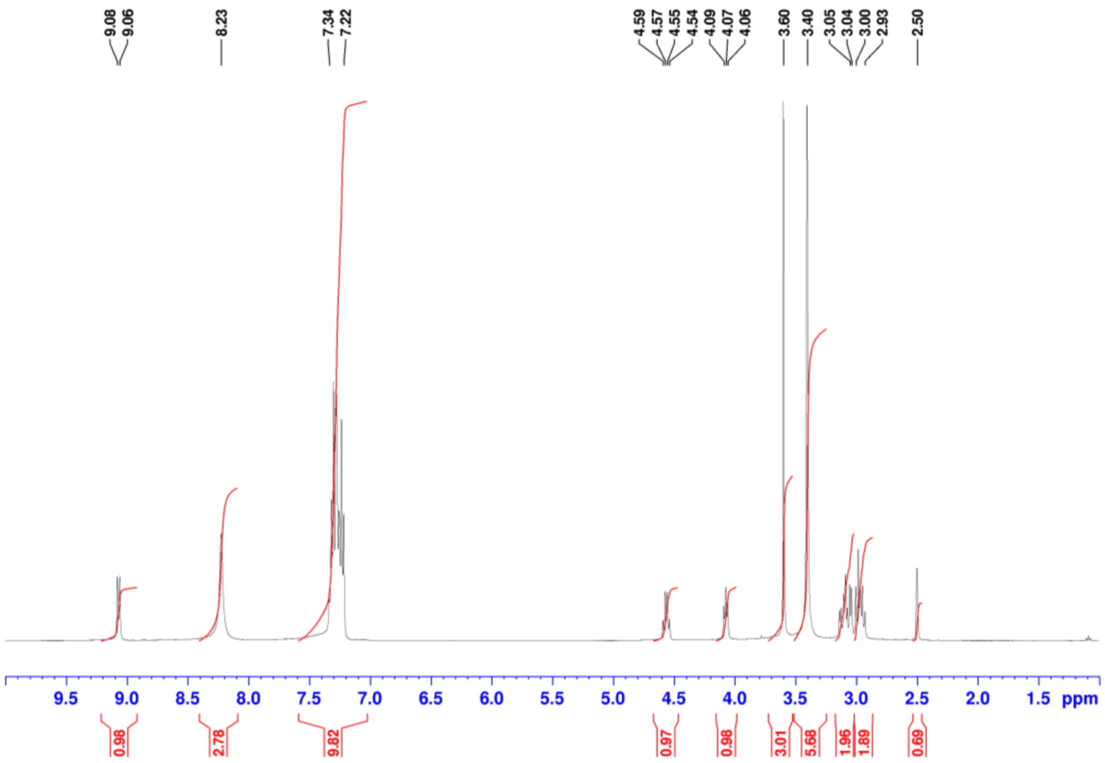

Figure S4. ^{1}H NMR spectrum of TFA·(L,L)-FFOMe in d_6-DMSO at 25 °C.

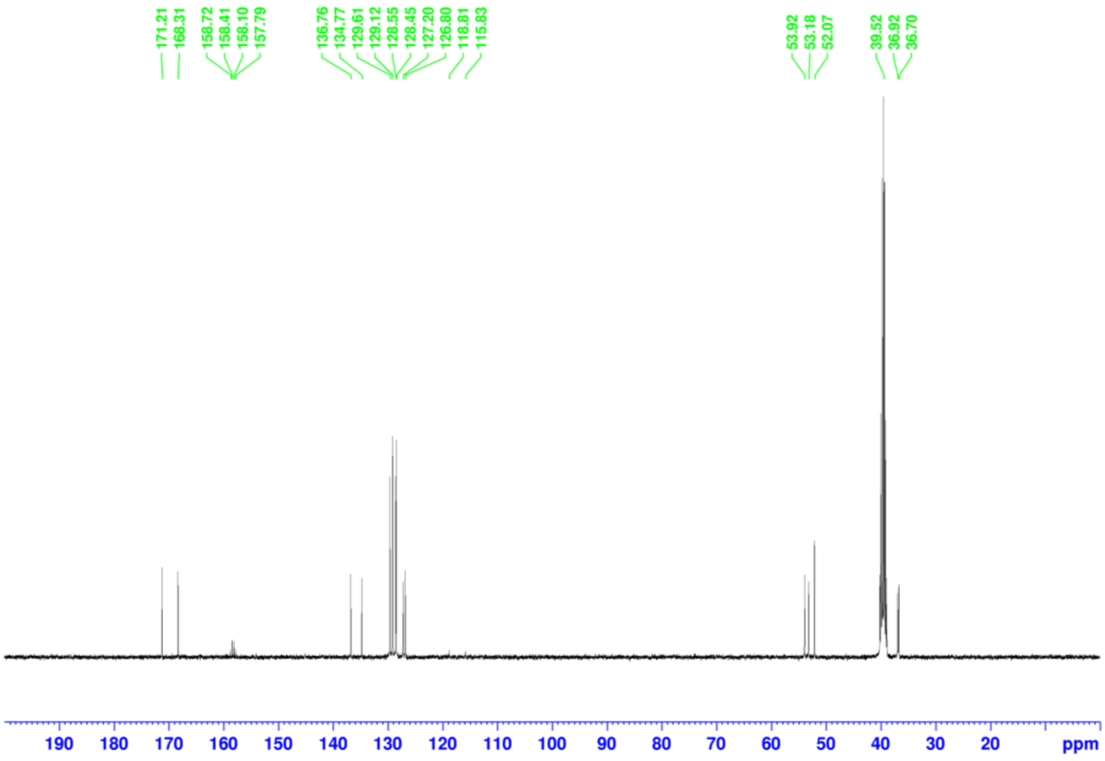

Figure S5. ^{13}C NMR spectrum of TFA·(L,L)-FFOMe in d_6-DMSO at 25 °C.

Methyl (2*S*)-2-[(2*S*)-2-[2-(naphthalen-2-yloxy)acetamido]-3-phenylpropanamido]-3-phenylpropanoate

To a solution of TFA·(L,L)-FFOMe (3.81 g, 8.64 mmol) in chloroform (30 mL) was added *N*-methylmorpholine (2 eq., 1.90 mL) and the mixture was stirred at room temperature for 10 min. Meanwhile, to a solution of 2-(naphthalene-2- acyloxy) acetic acid (1.75 g, 8.64 mmol) in chloroform (30 mL) was added *N*-methylmorpholine (2 eq., 1.90 mL) and isobutylchloroformate (1.1 eq., 1.24 mL) and the mixture was stirred on ice for 10 min. Subsequently, the former mixture was slowly added into the latter and kept stirring overnight on ice. The resulting clear solution was diluted with chloroform and washed in turn with 100 mL of 1M hydrochloric acid, water, and brine (saturated), before being dried (magnesium sulfate), and the solvent removed under vacuum. (L,L)-2NapFFOMe (3.65 g, 83%) was obtained as a light brown solid.

δ_H (400 MHz, DMSO-d_6, 25 °C) 8.61 (1H, d, *J* 7.55, N$\underline{H}$), 8.31 (0.7H, s, C$\underline{H}$ in chloroform), 8.14 (1H, d, *J* 8.56, N$\underline{H}$), 7.85-7.82 (2H, m, $\underline{H}_{Ar}$), 7.73 (1H, d, *J* 7.74, $\underline{H}_{Ar}$), 7.48-7.44 (1H, m, $\underline{H}_{Ar}$), 7.38-

7.34 (1.1H, m, $\underline{H}_{Ar}$), 7.28-7.12 (12.2H, m, $\underline{H}_{Ar}$), 4.67 (1H, td, *J* 9.16, 4.42, $C\underline{H}^*$), 4.55 (1.8H, s, $OC\underline{H}_2$), 4.54-4.50 (1.1H, m, $C\underline{H}^*$), 3.59 (3.1H, s, $OC\underline{H}_3$), 3.34 (4.9H, br s, $\underline{H}_2O$), 3.08-2.93 (3.2H, m, $PhC_a\underline{H}_2$ and $PhC_b\underline{H}_aH_b$), 2.86 (1H, dd, *J* 13.83, 9.43, $PhC_bH_a\underline{H}_b$), 2.50 (1.3H, quintet, residual DMSO-d_5). δ_C (100 MHz, DMSO-d_6, 25 °C) 171.75, 171.01, and 167.30 ($\underline{C}$=O), 155.52, 137.46, 136.98, 134.06, 129.39, 129.24, 129.09, 128.78, 128.30, 128.04, 127.54, 126.82, 126.62, 126.46, 126.33, 123.90, 118.49, and 107.34 ($\underline{C}_{Ar}$), 66.69 ($O\underline{C}H_2$), 53.69 ($\underline{C}H^*$), 53.23 ($\underline{C}H^*$), 51.90 ($O\underline{C}H_3$), 39.52 (septet, DMSO-d_6), 37.46 ($Ph\underline{C}_bH_2$), 36.65 ($Ph\underline{C}_aH_2$).

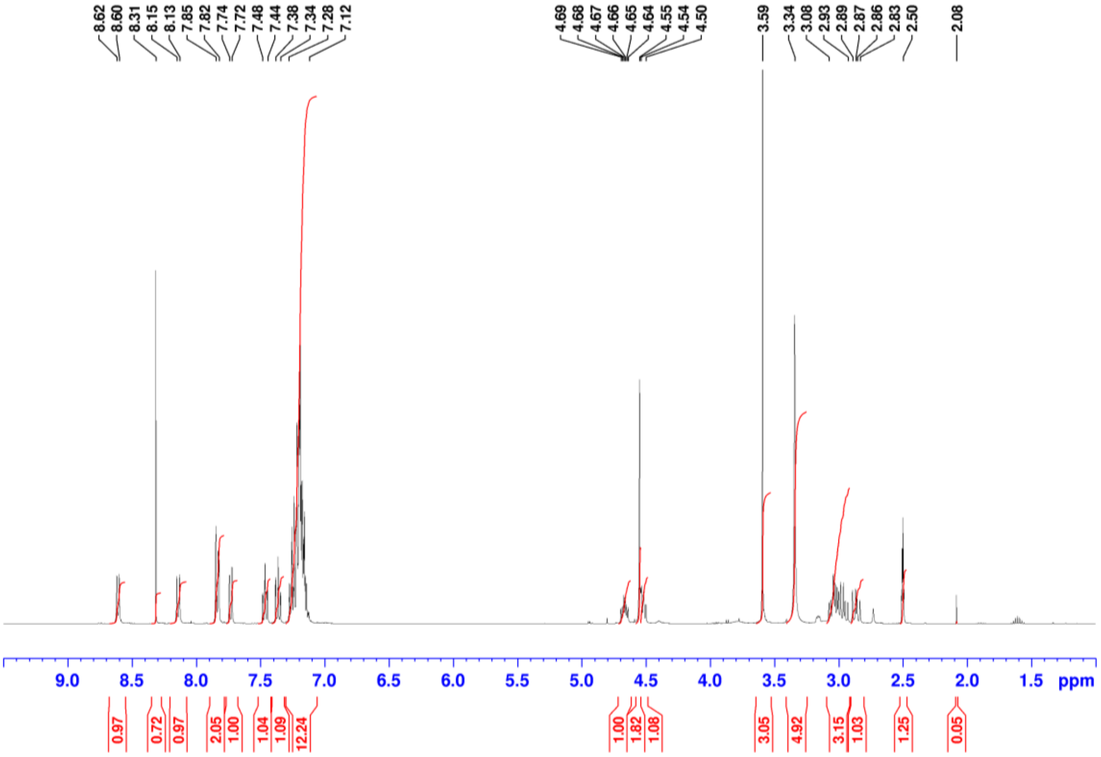

Figure S6. ^{1}H NMR spectrum of (L,L)-2NapFFOMe in d_6-DMSO at 25 °C.

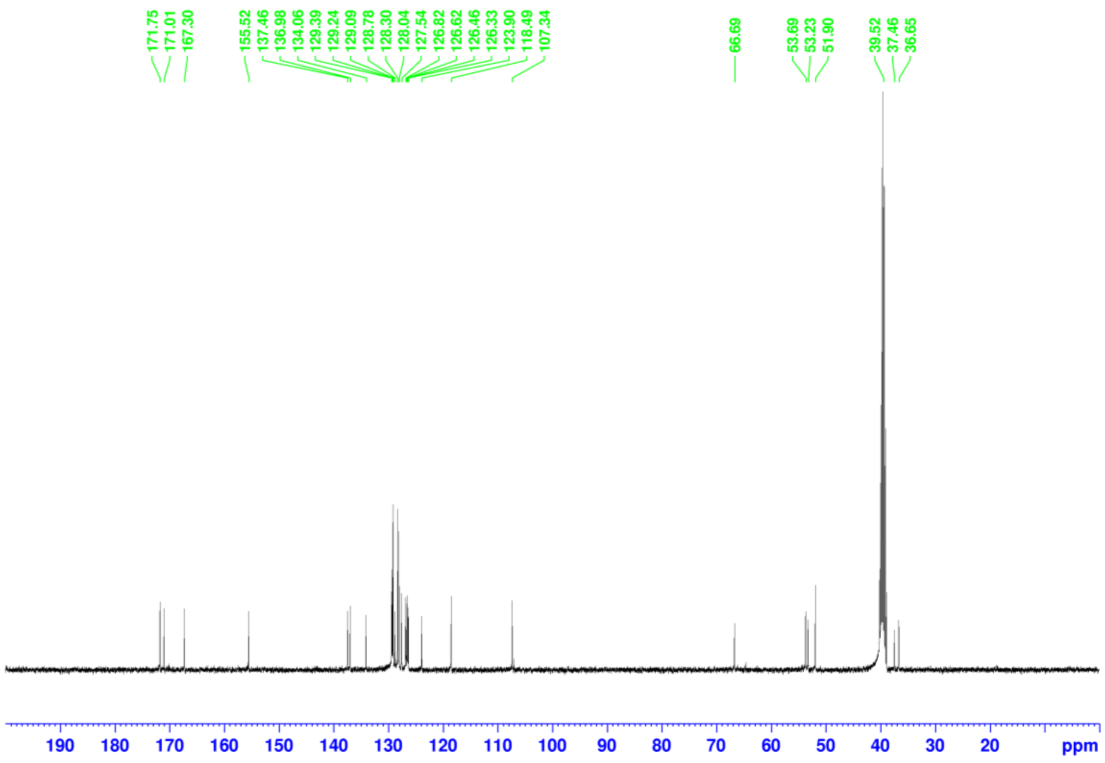

Figure S7. ^{13}C NMR spectrum of (L,L)-2NapFFOMe in d_6-DMSO at 25 °C.

<u>**(2*S*)-2-[(2*S*)-2-[2-(naphthalen-2-yloxy)acetamido]-3-phenylpropanamido]-3-phenylpropanoic acid**</u>

A solution of (L,L)-2NapFFOMe (3.65 g, 7.15 mmol) in tetrahydrofuran (30 mL) was stirred at room temperature for 10 min. Meanwhile, a solution of lithium hydroxide (685 mg, 28.59 mmol) in water (30 mL) prepared. The latter was slowly added into the former and the mixture stirred at room temperature for 1.5 h. TLC confirmed the absence of starting materials. The clear solution was poured into 200 mL 1 M hydrochloric acid and stirred overnight. The suspension was collected by filtration, followed by washing with 200 mL deionized water and a small amount of acetonitrile to remove the brown color. After that, 30 mL of acetonitrile was added and the mixture stirred for 1 hour before the solvent was removed under vacuum. 30 mL of diethyl ether was then added into the flask and then stirred overnight to help the bulk solids break up into small fragments. Finally, (L,L)-2NapFF (3.14 g, 88%) was obtained as a white powder by filtration.

δ_H (400 MHz, DMSO-d_6, 25 °C) 12.58 (0.4H, br s, COO<u>H</u>), 8.46 (1.0H, d, *J* 7.82, N<u>H</u>), 8.14 (1.0H, d, *J* 8.52, N<u>H</u>), 7.85-7.82 (1.9H, m, <u>H</u>$_{Ar}$), 7.73 (1.0H, d, *J* 8.20, <u>H</u>$_{Ar}$), 7.48-7.45 (1.0H, m, <u>H</u>$_{Ar}$), 7.38-

7.34 (1.1H, m, H$_{Ar}$), 7.27-7.12 (11.8H, m, H$_{Ar}$), 4.67 (1.0H, td, *J* 8.94, 3.72, CH*), 4.55 (1.8H, s, OCH$_2$), 4.52-4.47 (1.1H, m, CH*), 3.43 (8.5H, br s, H$_2$O), 3.11-3.02 (2.4H, m, PhCH$_2$), 2.97-2.84 (2.3H, m, PhCH$_2$), 2.50 (0.9H, quintet, residual DMSO-d$_5$). δ_C (100 MHz, DMSO-d$_6$, 25 °C) 172.79, 170.92, and 167.30 (C=O), 155.54, 137.55, 137.40, 134.08, 129.42, 129.31, 129.18, 128.81, 128.25, 128.04, 127.56, 126.85, 126.51, 126.49, 126.32, 123.92, 118.51, and 107.38 (C$_{Ar}$), 66.74 (OCH$_2$), 53.57 (CH*), 53.30 (CH*), 39.52 (septet, DMSO-d$_6$), 37.49 (PhCH$_2$), 36.75 (PhCH$_2$).

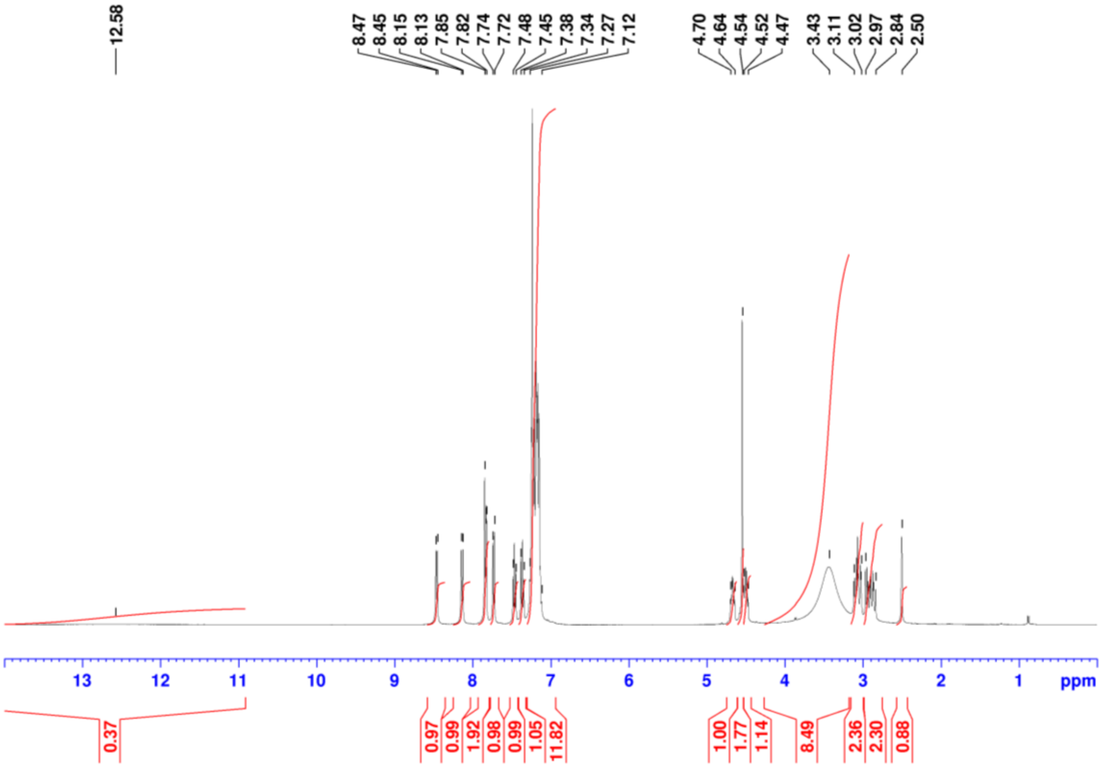

Figure S8. ^{1}H NMR spectrum of (L,L)-2NapFF in d$_6$-DMSO at 25 °C.

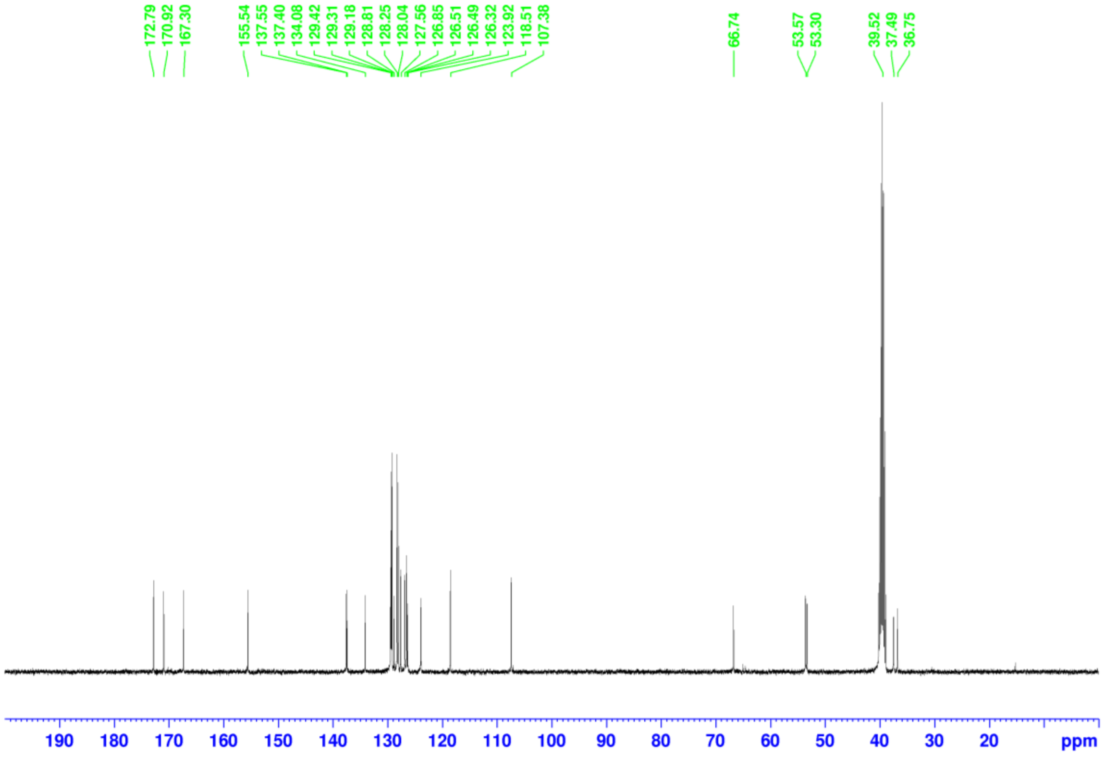

Figure S9. ^{13}C NMR spectrum of (L,L)-2NapFF in d_6-DMSO at 25 °C.

Synthesis of (L,D)-2NapFF

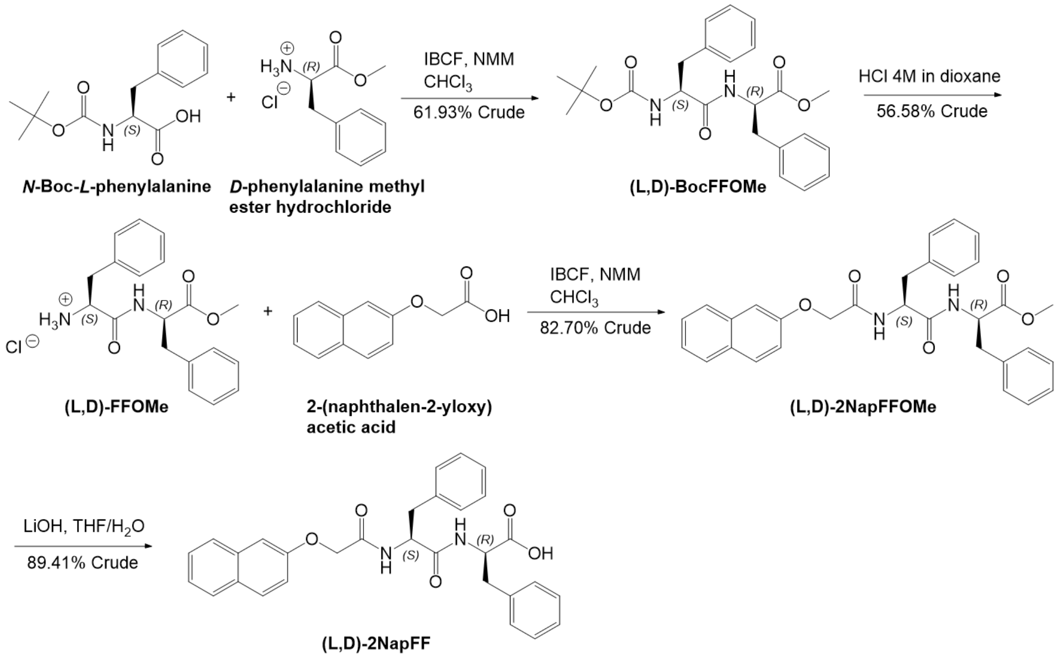

Figure 10. Synthesis route of (L,D)-2NapFF.

Methyl (2*R*)-2-[(2*S*)-2-{[(*tert*-butoxy)carbonyl]amino}-3-phenylpropanamido]-3-phenylpropanoate

To a solution of *N*-Boc-L-phenylalanine (2.65 g, 10 mmol) in chloroform (30 mL) was added *N*-methylmorpholine (2 eq., 2.20 mL) and isobutylchloroformate (1.1 eq., 1.43 mL) and the mixture was stirred on ice for 10 min. Meanwhile, to a solution of D-phenylalanine methyl ester hydrochloride (2.92 g, 10 mmol) in chloroform (30 mL) was added *N*-methylmorpholine (5 eq., 5.50 mL) and the mixture was stirred at room temperature for 10 min. The latter mixture was added to the former and stirred overnight on ice. The resulting clear solution was diluted with chloroform and washed in turn with 100 mL of 1M hydrochloric acid, water, and brine (saturated), before being dried (magnesium sulfate), and then the solvent was removed under vacuum. Afterwards, the yellowish powder was purified using column chromatography ((1:9 ethyl acetate/dichloromethane, ca. 50×4 cm, wet-loaded) and t (L,D)-BocFFOMe (2.64 g, 62%) was obtained as a white solid.

δ_H (400 MHz, DMSO-d_6, 25 °C) 8.49 (0.2H, d, *J* 7.30, Rot-1 N<u>H</u>CH*CO_2Me), 8.43 (0.8H, d, *J* 8.08, Rot-2 N<u>H</u>CH*CO_2Me), 7.29-7.13 (9.8H, m, $\underline{H}_{Ar}$), 6.73 (0.8H, d, *J* 8.81, Rot-2 N<u>H</u>Boc), 6.31 (0.2H, d, *J* 8.16, Rot-1 N<u>H</u>Boc), 4.55-4.50 (1H, m, C<u>H</u>*CO_2Me), 4.18 (0.8H, td, *J* 9.82, 4.00, Rot-2 C<u>H</u>*NHBoc), approx. 4.07-4.02 (0.2H, m, Rot-1 C<u>H</u>*NHBoc), 3.63 (2.9H, s, OC$\underline{H}_3$), 3.33 (8.3H, br s, $\underline{H}_2$O), 3.06 (1H, dd, *J* 13.68, 5.13, PhC$\underline{H}_a$$H_b$CH*$CO_2$Me), 2.88 (1H, dd, *J* 13.67, 9.48, PhCH$_a$$\underline{H}_b$CH*$CO_2$Me), 2.69 (0.8H, dd, *J* 13.76, 3.88, PhC$\underline{H}_a$$H_b$CH*NHBoc), 2.61-2.39 (0.9H, m, PhCH$_a$$\underline{H}_b$CH*NHBoc overlapped by residual DMSO-d_5 peak), 1.28 (7.1H, s, Rot-2 C(C$\underline{H}_3$)$_3$), 1.21 (1.6H, s, Rot-1 C(C$\underline{H}_3$)$_3$)). δ_C (100 MHz, DMSO-d_6, 25 °C) 171.86, 171.62, and 155.07 (<u>C</u>=O), 138.01, 137.05, 129.19, 129.15, 128.21, 127.90, 126.58, and 126.10 ($\underline{C}_{Ar}$), 77.93 (<u>C</u>(CH_3)$_3$), 55.30 (<u>C</u>H*NHBoc), 53.36 (<u>C</u>H*CO_2Me), 51.92 (O<u>C</u>H_3), 39.52 (septet, DMSO-d_6), 37.52 (Ph<u>C</u>H_2CH*NHBoc), 36.96(Ph<u>C</u>H_2CH*CO_2Me), 28.11 (Rot-2 C(<u>C</u>H_3)$_3$), 27.73 (Rot-1 C(<u>C</u>H_3)$_3$).

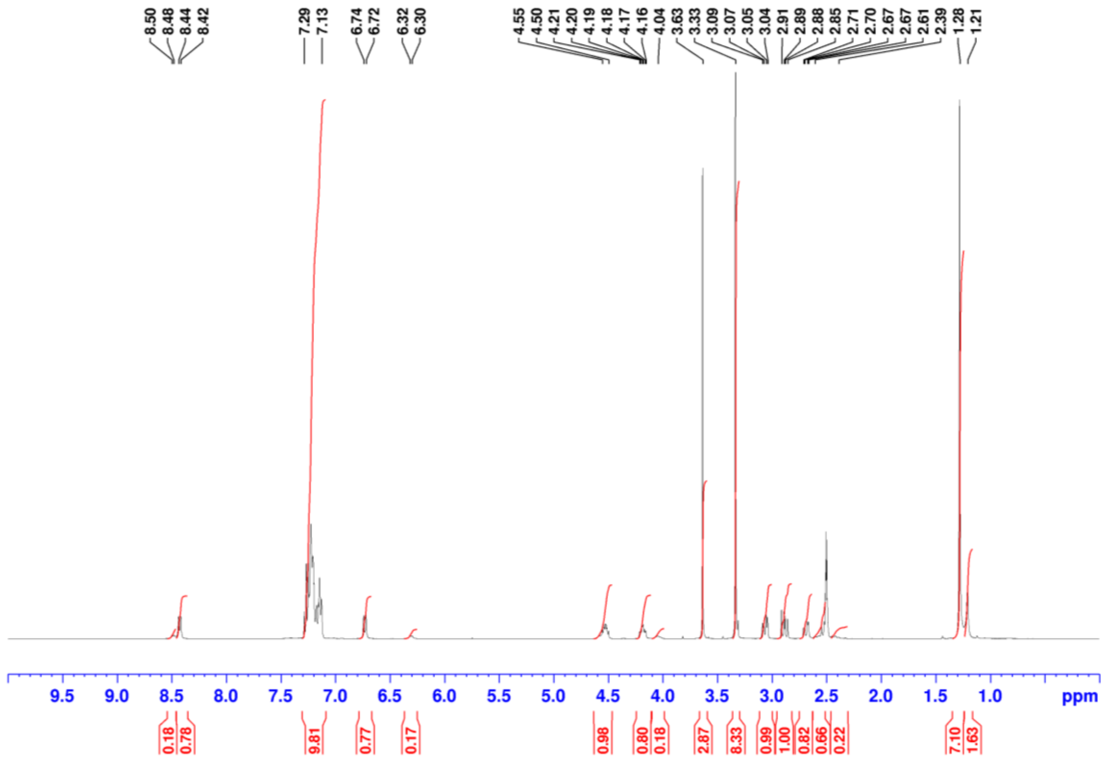

Figure S11. ^{1}H NMR spectrum of (L,D)-BocFFOMe in d_6-DMSO at 25 °C.

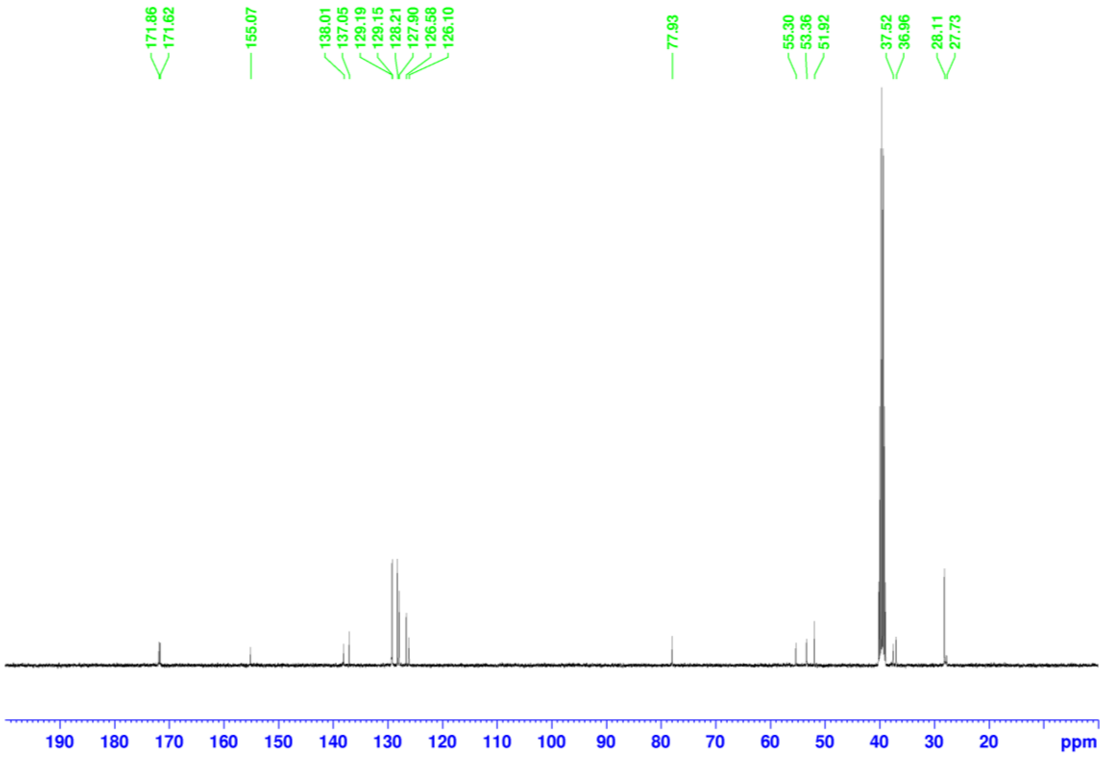

Figure S12. ^{13}C NMR spectrum of (L,D)-BocFFOMe in d_6-DMSO at 25 °C.

<u>Methyl</u>

<u>(2*R*)-2-[(2*S*)-2-azaniumyl-3-phenylpropanamido]-3-phenylpropanoate hydrochloride</u>

To a solution of (L,D)-BocFFOMe (2.64 g, 6.19 mmol) in 1,4-dioxane (15 mL) was added hydrogen chloride 4M in 1,4-dioxane (30.96 mL, ca. 20 eq.) and the mixture stirred overnight at room temperature. TLC confirmed the absence of starting materials. The resulted mixture was evaporated in vacuo, redissolved in 30 mL acetonitrile, well-stirred and then the solvent removed under vacuum. (L,D)-FFOMe (1.27 g, 57%) was collected as a white foam.

δ_H (400 MHz, DMSO-d_6, 25 °C) 9.30 (0.9H, d, *J* 8.07, Rot-2 N$\underline{H}$), 9.11 (0.1H, d, *J* 8.08, Rot-1 N$\underline{H}$), 8.27 (2.9H, br s, N$\underline{H}_3^+$), 7.30-7.21 (8.0H, m, $\underline{H}_{Ar}$), 7.03-6.97 (2.0H, m, $\underline{H}_{Ar}$), 4.56-4.50 (1H, m, NHC$\underline{H}^*$), 4.10 (1H, t, *J* 6.38, C$\underline{H}^*$$NH_3^+$), 3.63 (2.7H, s, OC$\underline{H}_3$), 3.56 (0.3H, s, C$\underline{H}_2$ in 1,4-dioxane), 3.38 (6.5H, br s, $\underline{H}_2$O), 3.09-2.93 (2.0H, m, PhC$\underline{H}_2$), 2.88-2.72 (2H, m, PhC$\underline{H}_2$), 2.50 (1.0H, quintet, residual DMSO-d_5), 2.07 (0.3H, s, C$\underline{H}_3$ in acetonitrile). δ_C (100 MHz, DMSO-d_6, 25 °C), not all carbons resolved) 171.42 and 167.98 ($\underline{C}$=O), 136.86, 134.60, 129.62, 129.25, 128.35, 127.02, and 126.78 ($\underline{C}_{Ar}$), 53.79 (NH$\underline{C}$H*), 53.10 ($\underline{C}$H*NH^{3+}), 52.08 (O$\underline{C}H_3$), 39.52 (septet, DMSO-d_6), 36.88 and 36.66 (Ph$\underline{C}H_2$).

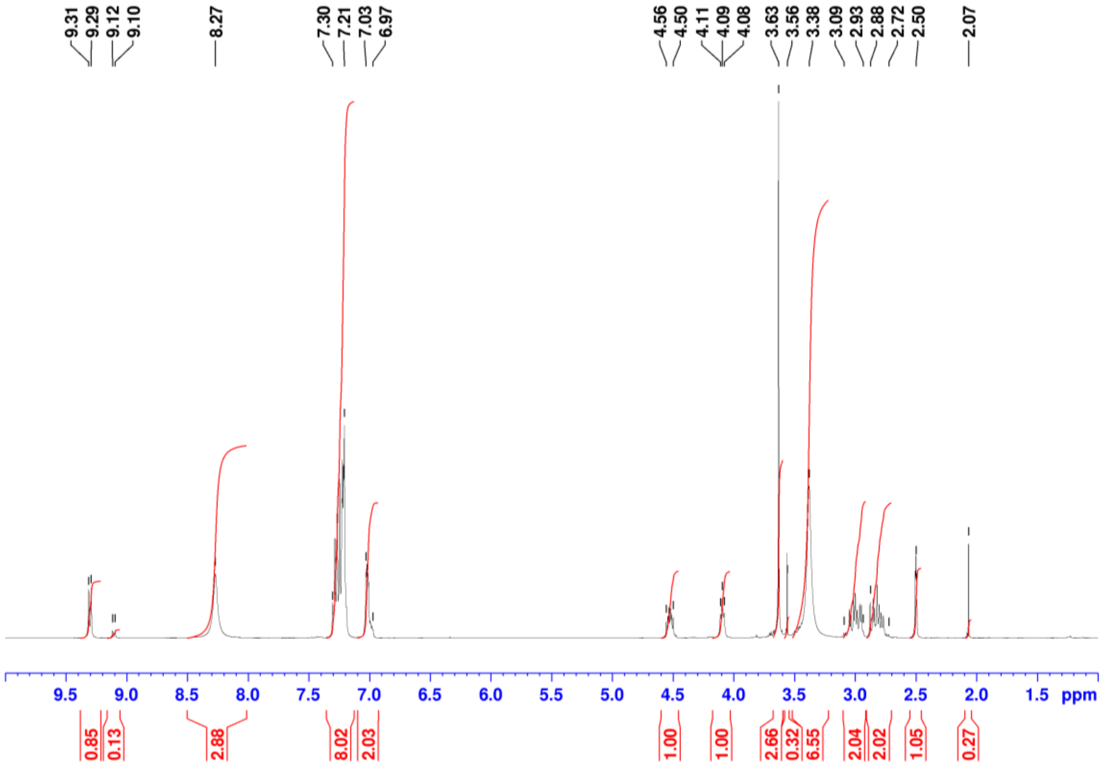

Figure S13. ^{1}H NMR spectrum of (L,D)-FFOMe in d_6-DMSO at 25 °C.

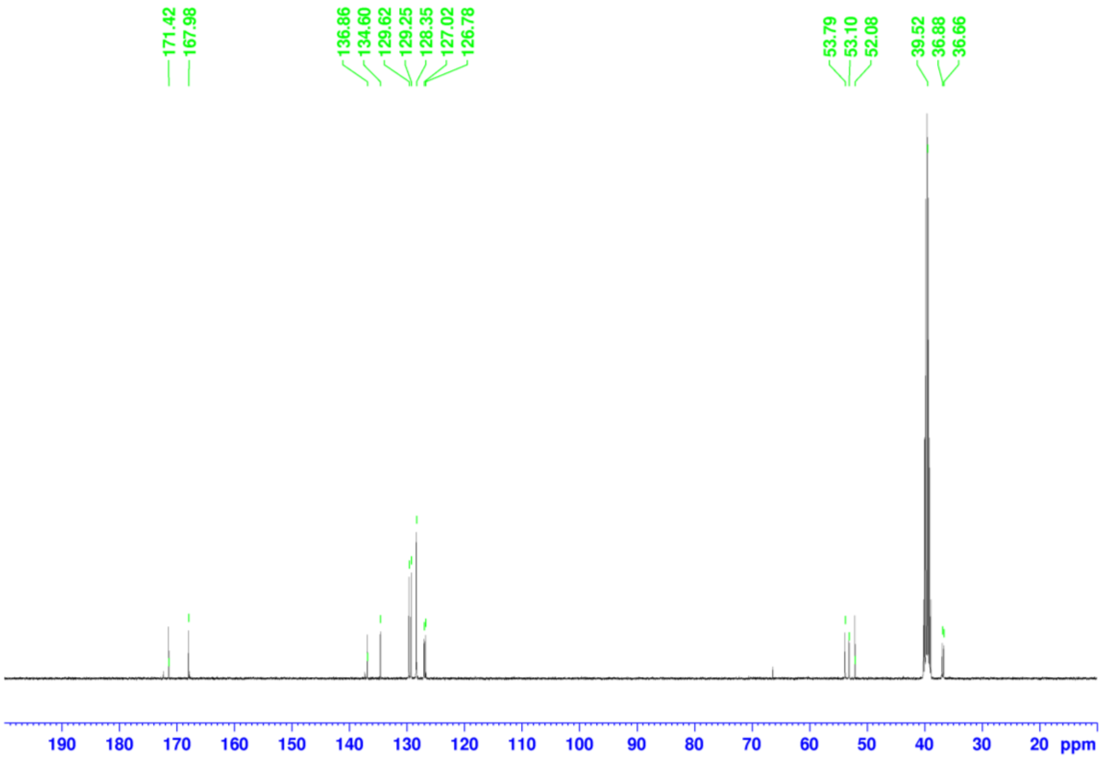

Figure S14. ^{13}C NMR spectrum of (L,D)-FFOMe in d_6-DMSO at 25 °C.

Methyl (2*R*)-2-[(2*S*)-2-[2-(naphthalen-2-yloxy)acetamido]-3-phenylpropanamido]-3-phenylpropanoate

To a solution of (L,D)-FFOMe (1.27 g, 4.61 mmol) in chloroform (30 mL) was added *N*-methylmorpholine (2 eq., 1.90 mL) and the mixture was stirred at room temperature for 10 min. To a solution of 2-(naphthalene-2-acyloxy) acetic acid (0.93 g, 4.61 mmol) in chloroform (30 mL) was added *N*-methylmorpholine (2 eq., 1.90 mL) and isobutylchloroformate (1.1 eq., 1.24 mL), and the mixture was stirred on ice for 10 min. Subsequently, the former mixture was slowly added into the latter and stirred overnight on ice. The resulting clear solution was diluted with chloroform and washed in turn with 100 mL of 1M hydrochloric acid, water, and brine (saturated), dried (magnesium sulfate), and the solvent removed under vacuum. The resulting yellowish powder was purified using column chromatography ((1:9 ethyl acetate/dichloromethane, ca. 50×4 cm, wet-loaded) and (L,D)-2NapFFOMe (1.94 g, 83%) was obtained as a white powder.

δ_H (400 MHz, DMSO-d$_6$) 8.74 (0.9H, d, *J* 8.14, NH), 8.11 (0.9H, d, *J* 8.59, NH), 7.83 (1.8H, d, *J* 8.83, H$_{Ar}$), 7.73 (1.0H, d, *J* 8.19, H$_{Ar}$), 7.47 (0.9H, t, *J* 7.64, H$_{Ar}$), 7.37 (1.0H, t, *J* 7.92, H$_{Ar}$), 7.30-7.00 (12.1H, m, H$_{Ar}$), 4.66 (1H, td, *J* 8.91, 4.43, CH*), 4.56 (1.8H, s, OCH$_2$), 4.54-4.51 (0.9H, m, CH*), 3.62 (2.8H, s, OCH$_3$), 3.41 (9.7H, br s, H_2O), 3.09 (1.1H, dd, *J* 13.59, 4.88, PhCH$_2$), 2.87 (1H, dd, *J* 13.52, 10.14, PhCH$_2$), 2.76 (0.9H, dd, *J* 13.56, 3.99, PhCH$_2$), 2.66 (1.0H, dd, *J* 13.50, 9.33, PhCH$_2$), 2.50 (0.9H, quintet, residual DMSO-d$_5$). δ_C (100 MHz, DMSO-d$_6$, 25 °C) 171.95, 170.85, and 167.27 (C=O), 155.52, 137.33, 137.14, 134.08, 129.42, 129.30, 129.21, 128.79, 128.31, 128.00, 127.56, 126.83, 126.71, 126.49, 126.31, 123.92, 118.50, and 107.33 (C$_{Ar}$), 66.68 (OCH$_2$), 53.59 (CH*), 53.30 (CH*), 52.05 (OCH$_3$), 39.52 (septet, DMSO-d$_6$), 37.73 (PhCH$_2$), 36.96 (PhCH$_2$).

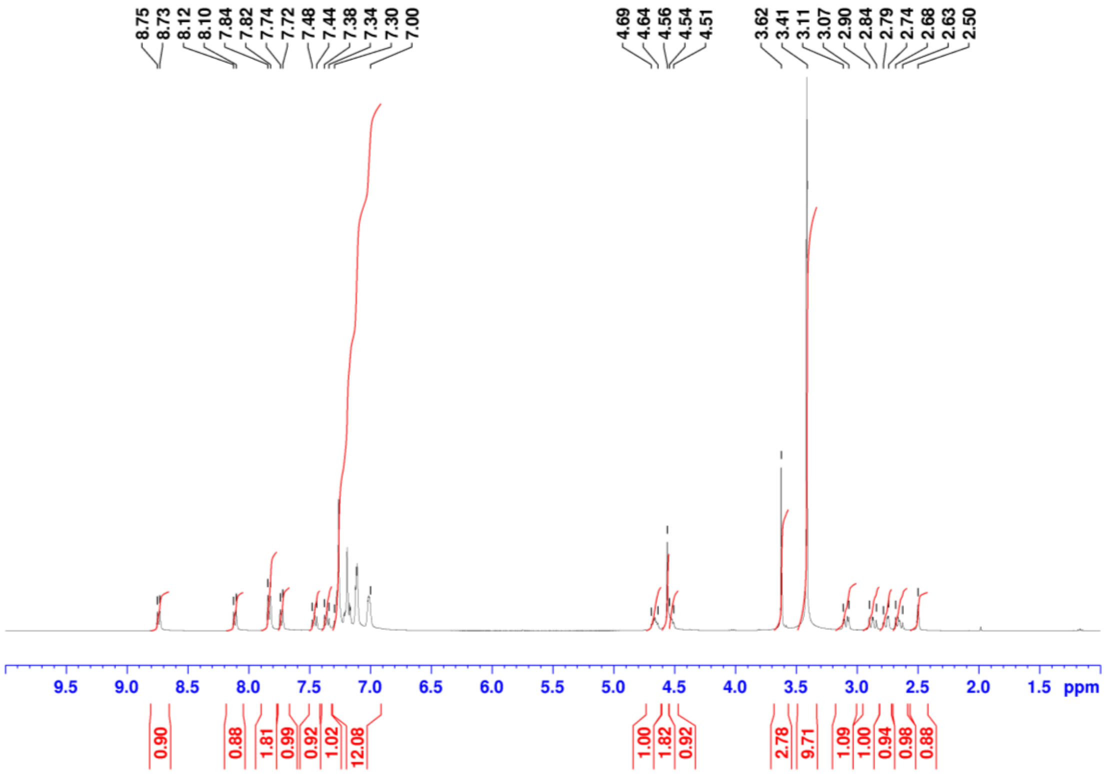

Figure S15. ^{1}H NMR spectrum of (L,D)-2NapFFOMe in d$_6$-DMSO at 25 °C.

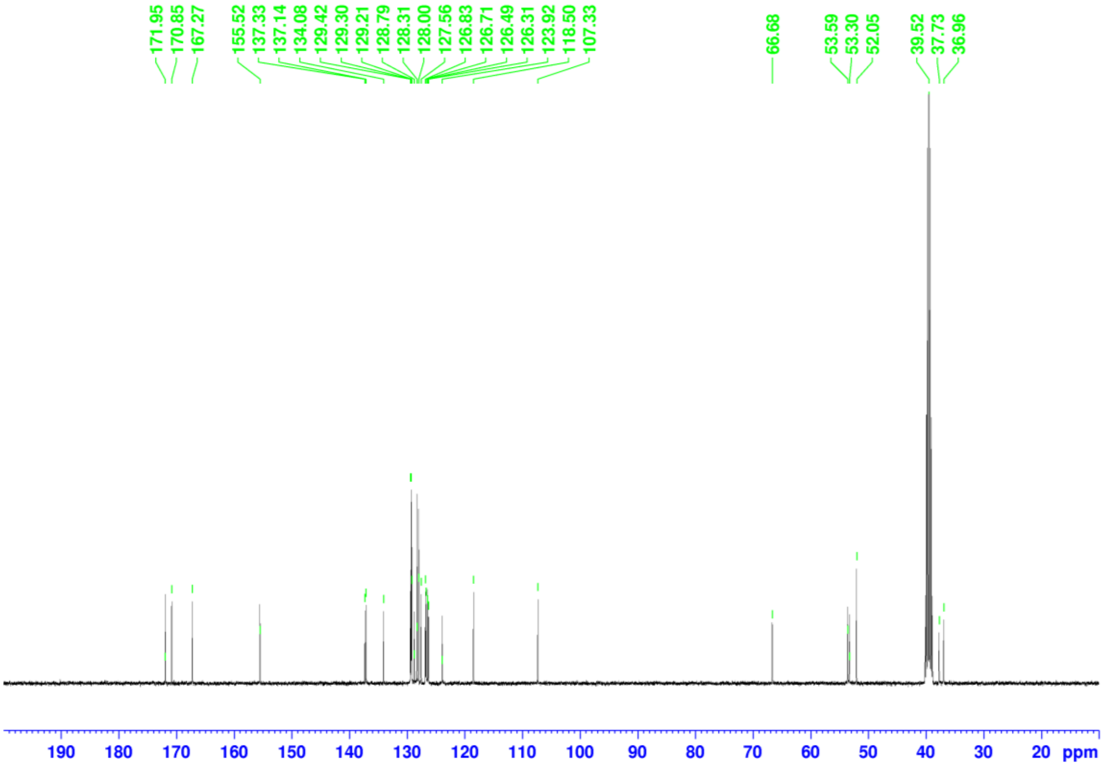

Figure S16. ^{13}C NMR spectrum of (L,D)-2NapFFOMe in d$_6$-DMSO at 25 °C.

(2*R*)-2-[(2*S*)-2-[2-(naphthalen-2-yloxy)acetamido]-3-phenylpropanamido]-3-phenylpropanoic acid

A solution of (L,D)-2NapFFOMe (1.94 g, 3.81 mmol) in tetrahydrofuran (30 mL) was stirred at room temperature for 10 minutes. A solution of lithium hydroxide (374 mg, 20 eq.) in water (30 mL) was slowly added into the former solution and kept stirring at room temperature for 1 h. TLC confirmed the absence of starting materials. The resulting clear solution was poured into 200 mL of 1 M hydrochloric acid and stirred overnight to allow total precipitate. Then the suspension was collected by filtration using a Buchner funnel, washed with 200 mL deionized water and then a small amount of acetonitrile to remove the brown color. The obtained solid was further dried under vacuum. (L,D)-2NapFF (1.69 g, 89 %) was collected as a white solid.

δ_H (400 MHz, DMSO-d$_6$) 12.90 (0.8H, br s, COOH), 8.63 (1.0H, d, *J* 8.32, NH), 8.10 (1.0H, d, *J* 8.56, NH), 7.83 (2.0H, d, *J* 8.72, H$_{Ar}$), 7.74 (1.0H, d, *J* 8.20, H$_{Ar}$), 7.46 (1.1H, t, *J* 7.20, H$_{Ar}$), 7.36 (1.1H, t, *J* 7.28, H$_{Ar}$), 7.29-6.99 (11.8H, m, H$_{Ar}$), 4.69 (1H, td, *J* 8.88, 4.28, CH*), 4.57 (1.8H, s, OCH$_2$), 4.54-4.49 (1.0H, m, CH*), 3.43 (1.4H, br s, H$_2$O), 3.14 (1.1H, dd, *J* 13.62, 4.41, Ph$_a$CH$_a$H$_b$),

2.87 (1.0H, dd, *J* 13.56, 10.19, $\mathrm{Ph_aCH_a\underline{H}_b}$), 2.78 (1.0H, dd, *J* 13.60, 3.94, $\mathrm{Ph_bC\underline{H}_aH_b}$), 2.65 (1.0H, dd, *J* 13.57, 9.24, $\mathrm{Ph_bCH_a\underline{H}_b}$), 2.50 (0.9H, quintet, residual DMSO-d_5). δ_C (100 MHz, DMSO-d_6, 25 °C) 172.92, 170.65, and 167.18 ($\underline{\mathrm{C}}$=O), 155.52, 137.54, 137.35, 134.07, 129.41, 129.32, 129.22, 128.79, 128.23, 127.95, 127.54, 126.83, 126.57, 126.47, 126.24, 123.90, 118.47, and 107.36 ($\underline{\mathrm{C}}_{\mathrm{Ar}}$), 66.72 ($\mathrm{O\underline{C}H_2}$), 53.55 ($\underline{\mathrm{C}}$H*), 53.30 ($\underline{\mathrm{C}}$H*), 39.52 (septet, DMSO-d_6), 37.81 ($\mathrm{Ph_b\underline{C}H_2}$), 37.07 ($\mathrm{Ph_a\underline{C}H_2}$).

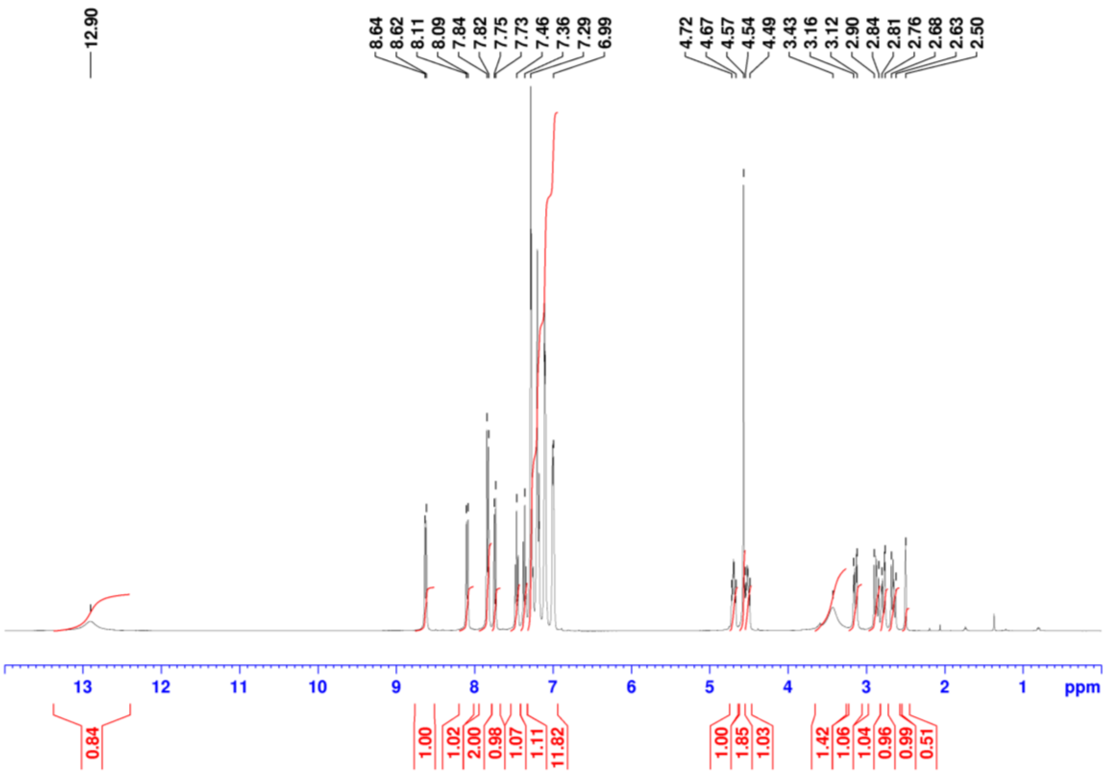

Figure S17. ^{1}H NMR spectrum of (L,D)-2NapFF in d_6-DMSO at 25 °C.

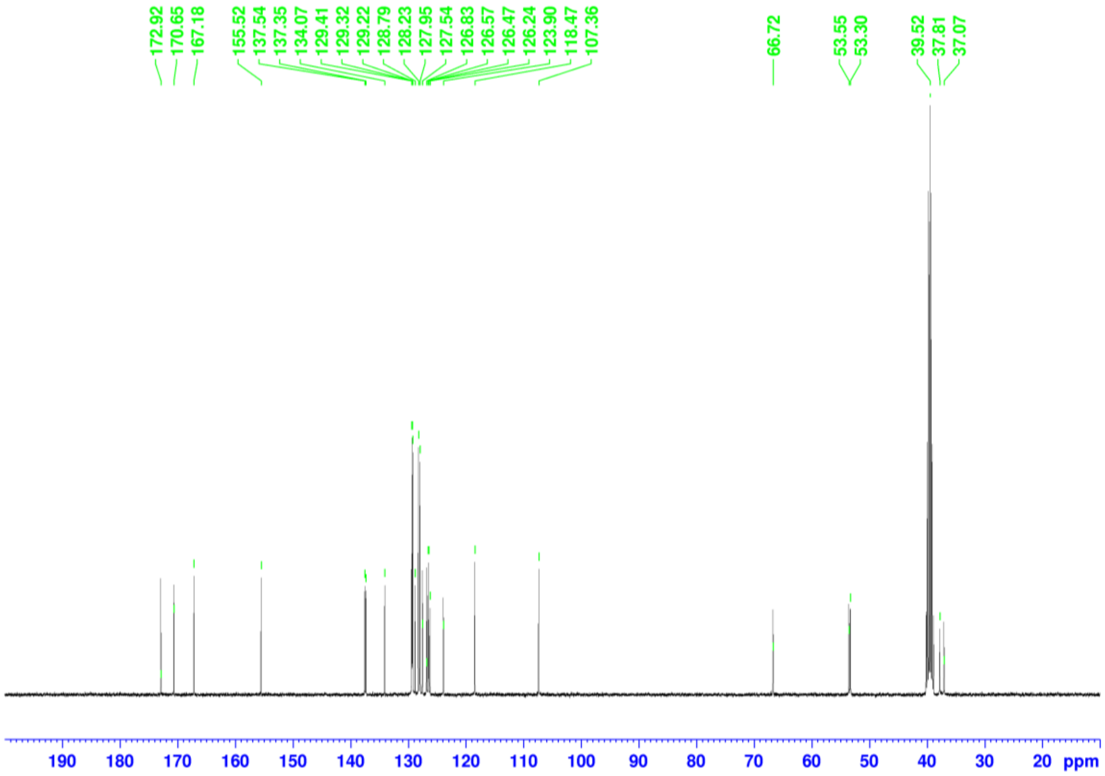

Figure S18. ^{13}C NMR spectrum of (L,D)-2NapFF in d_6-DMSO at 25 °C.

Additional Figures

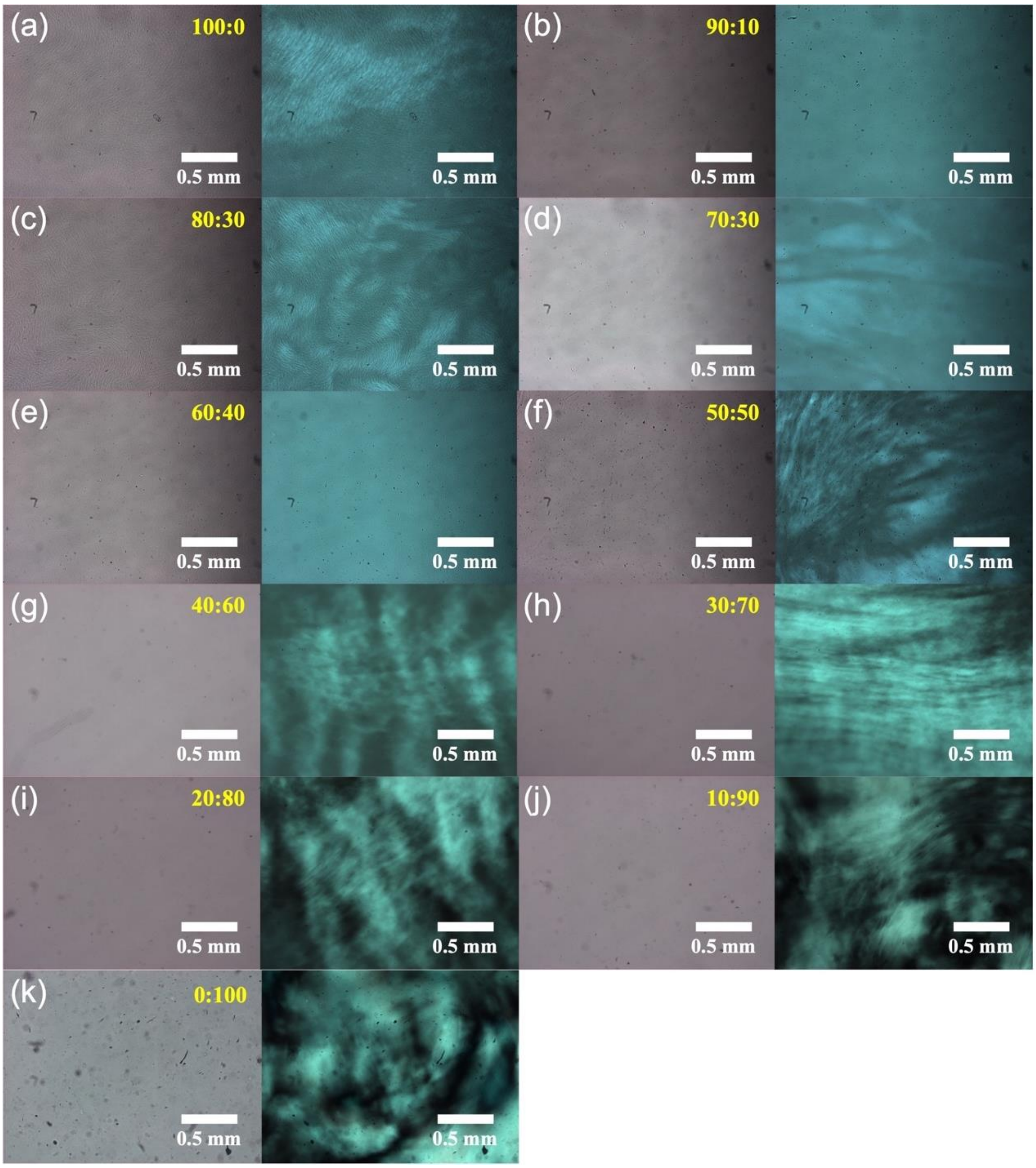

Figure S19. Optical microscopy (5×magnification) of binary system solutions with volume ratios from 100:0 to 0:100 of 10 mg/mL (L,L)-, (L,D)-2NapFF stock solutions. Images were taken with no polarizers (NP) or cross-polarizers (CP).

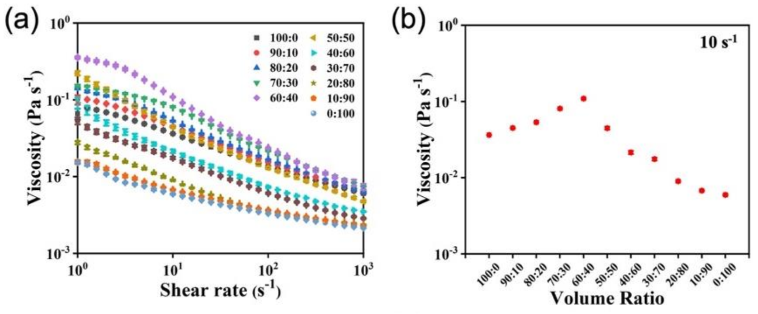

Figure S20. (a) The viscosity of binary system solutions with volume ratios from 100:0 to 0:100 of 10 mg/mL (L,L)-, (L,D)-2NapFF stock solutions. (b) Plot show viscosity at a shear rate of 10 s^{-1} against volume ratio.

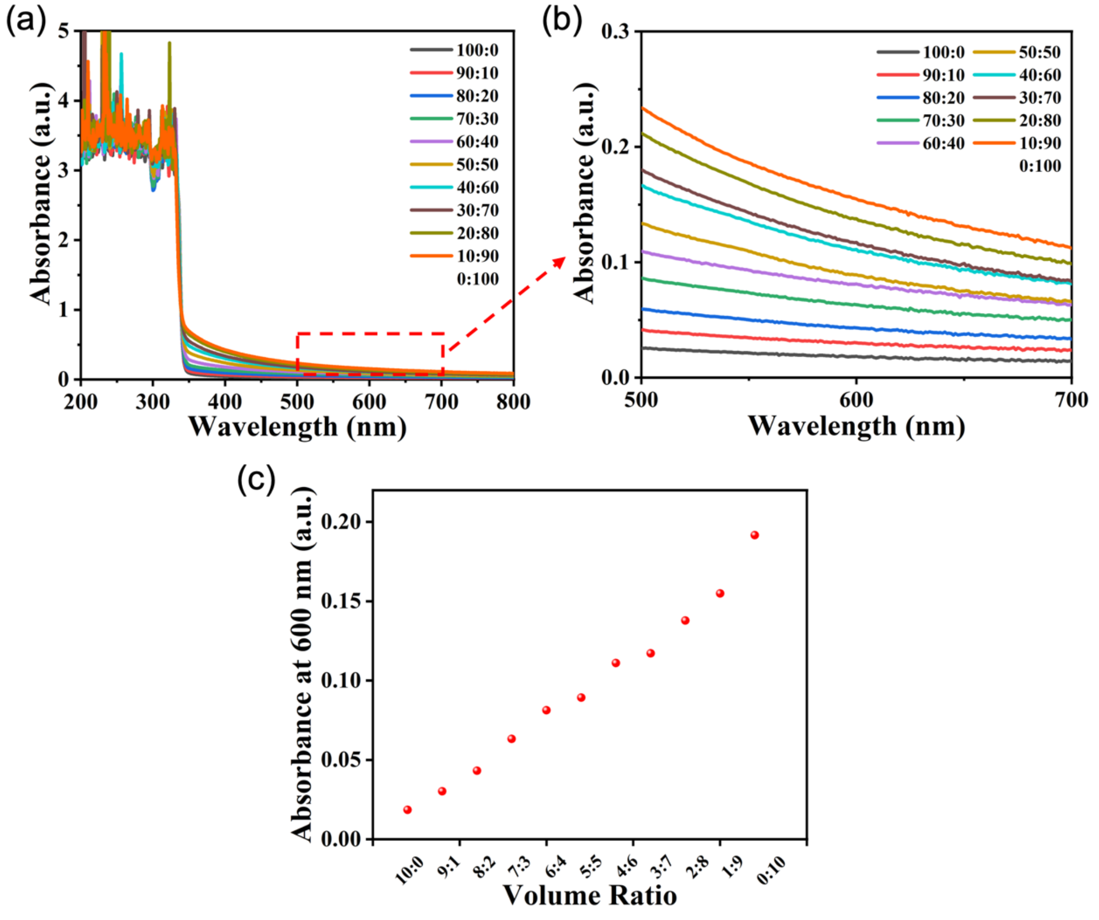

Figure S21. (a) UV-Vis absorption spectra of solutions containing 10 mg/mL of (L,L)- and (L,D)-2NapFF at ratios ranging from 100:0 to 0:100 measured using a 2 mm path length quartz cuvette at 25 °C. (b) Spectra depicted the enlarged regions of the graph (a) at wavelengths of 500-700 nm. (c) Change in turbidity over composition at 600 nm for solutions.

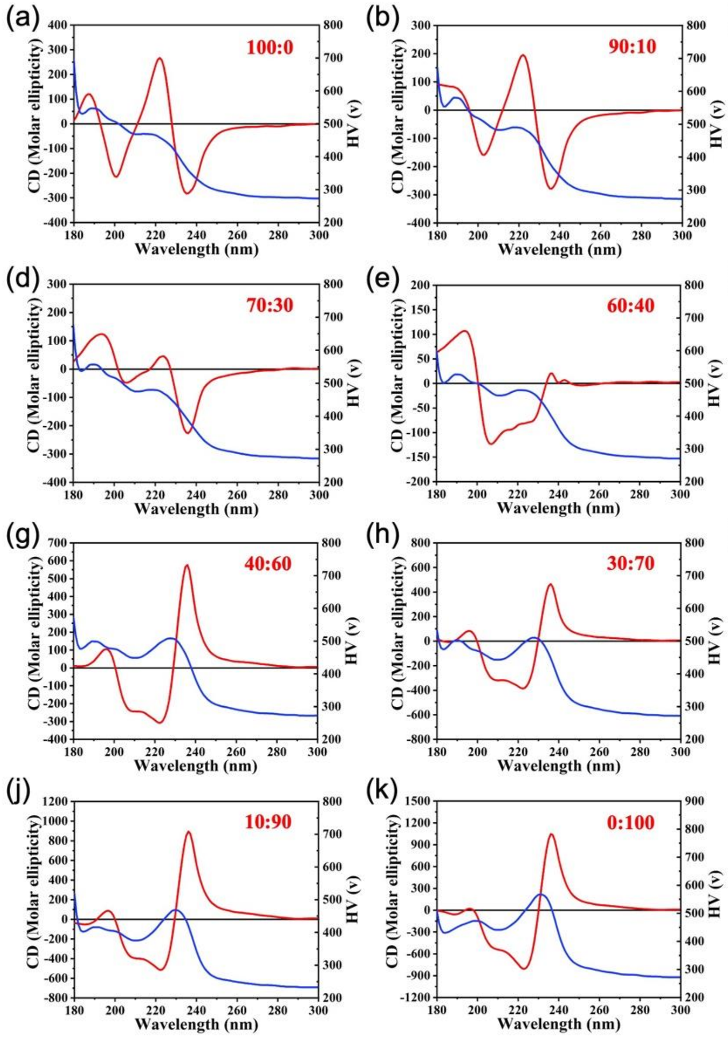

Figure S22. HT and CD data for binary system solutions with volume ratios from 100:0 to 0:100 of 10 mg/mL (L,L)-, (L,D)-2NapFF stock solutions.

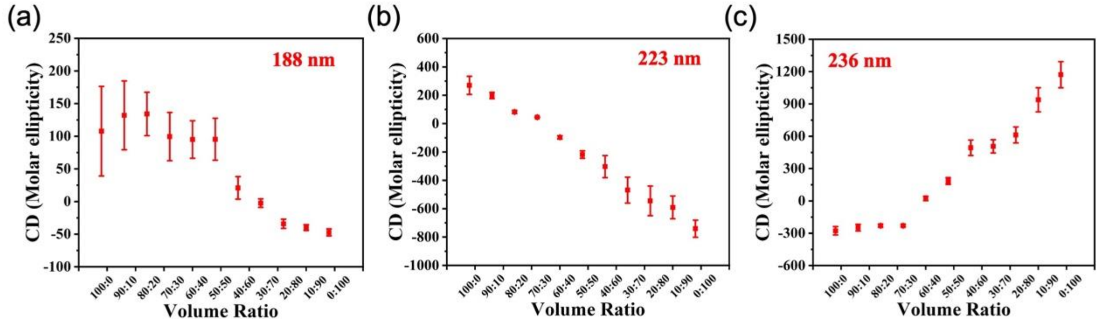

Figure S23. Plot of CD signal intensity at (a) 188 nm, (b) 223 nm, and (c) 236 nm against ratio at for binary system solutions with volume ratios from 100:0 to 0:100 of 10 mg/mL (L,L)-, (L,D)-2NapFF stock solutions.

Table S1. CD data at 188 nm for binary system solutions with volume ratios from 100:0 to 0:100 of 10 mg/mL (L,L)-, (L,D)-2NapFF stock solutions.

Volume Ratio	1	2	3	4	5	6	7	8	Average	Error bar
100:0	77.5	66.2	102.2	59.6	23.7	261.0	153.2	119.2	107.8	68.7
90:10	163.7	162.2	49.0	91.5	206.5	82.5	189.7	111.8	132.1	52.7
80:20	175.9	178.8	153.9	111.1	90.6	143.7	87.7	131.5	134.2	33.2
70:30	85.3	69.6	108.9	86.9	97.1	192.2	74.7	81.3	99.5	36.9
60:40	89.5	133.0	87.0	72.3	121.4	92.0	124.6	40.7	95.1	28.7
50:50	52.3	66.1	147.1	113.6	88.2	132.6	62.3	101.0	95.4	32.2
40:60	54.0	5.3	1.9	8.6	21.4	9.1	27.0	39.6	20.9	17.3
30:70	-6.7	-2.4	3.6	-2.5	11.6	-9.3	-5.7	-8.5	-2.5	6.6
20:80	-25.2	-29.9	-45.6	-34.7	-32.2	-39.5	-24.7	-41.0	-34.1	7.0
10:90	-34.3	-37.8	-41.2	-43.8	-32.6	-46.6	-40.2	-42.3	-39.9	4.4
0:100	-38.8	-43.2	-43.8	-47.6	-54.2	-45.9	-49.1	-56.0	-47.3	5.4

Table S2. CD data at 223 nm of binary system solutions with volume ratios from 100:0 to 0:100 of 10 mg/mL (L,L)-, (L,D)-2NapFF stock solutions.

Volume Ratio	1	2	3	4	5	6	7	8	Average	Error bar
100:0	188.2	278.3	271.8	279.2	177.2	394.5	312.2	257.0	269.8	64.1
90:10	195.4	179.8	164.2	200.2	195.2	188.4	239.4	222.6	198.1	22.1
80:20	111.0	77.7	82.6	80.2	74.1	75.9	75.8	78.5	82.0	11.3
70:30	41.3	43.2	42.9	46.7	44.3	46.0	43.5	48.9	44.6	2.3
60:40	-81.5	-115.9	-91.2	-99.4	-99.9	-100.2	-105.9	-79.5	-96.7	11.4
50:50	-194.8	-188.7	-223.7	-213.0	-195.1	-258.2	-212.4	-260.1	-218.3	26.0
40:60	-313.3	-225.2	-232.1	-231.5	-306.0	-264.8	-433.1	-419.5	-303.2	77.7
30:70	-518.7	-383.1	-310.5	-469.1	-575.2	-477.6	-603.0	-414.5	-469.0	91.7
20:80	-507.9	-446.1	-661.6	-480.6	-578.8	-666.9	-366.6	-648.2	-544.6	104.5
10:90	-645.2	-573.6	-512.0	-664.3	-458.7	-611.9	-544.2	-717.0	-590.9	79.7
0:100	-731.2	-732.7	-830.1	-779.9	-638.4	-672.5	-737.0	-807.2	-741.1	60.4

Table S3. CD data at 226 nm of binary system solutions with volume ratios from 100:0 to 0:100 of 10 mg/mL (L,L)-, (L,D)-2NapFF stock solutions.

Volume Ratio	1	2	3	4	5	6	7	8	Average	Error bar
100:0	-224.6	-277.4	-237.2	-248.7	-291.2	-351.4	-303.7	-281.5	-277.0	38.1
90:10	-220.3	-223.4	-222.4	-232.4	-222.3	-278.8	-296.1	-286.2	-247.8	30.9
80:20	-262.5	-213.8	-234.9	-231.5	-215.3	-231.7	-219.4	-233.1	-230.3	14.5
70:30	-210.5	-235.0	-233.4	-216.7	-226.9	-240.3	-237.2	-239.9	-230.0	10.4
60:40	20.2	59.1	55.1	6.2	17.8	8.3	8.7	11.4	23.4	20.0
50:50	172.2	148.7	179.7	174.2	142.7	214.9	238.4	205.6	184.5	30.8
40:60	333.2	567.8	464.2	517.4	577.1	493.7	524.7	471.4	493.7	71.7
30:70	578.5	465.6	389.7	467.4	536.4	497.2	586.7	529.3	506.4	61.2
20:80	521.2	530.0	557.0	574.3	745.6	680.2	651.3	641.4	612.6	74.4
10:90	1086.9	727.5	894.5	1009.0	1068.1	891.7	974.2	858.4	938.8	111.9
0:100	1149.5	1121.9	1370.6	1360.9	1197.9	1058.0	1068.7	1045.9	1171.7	121.6

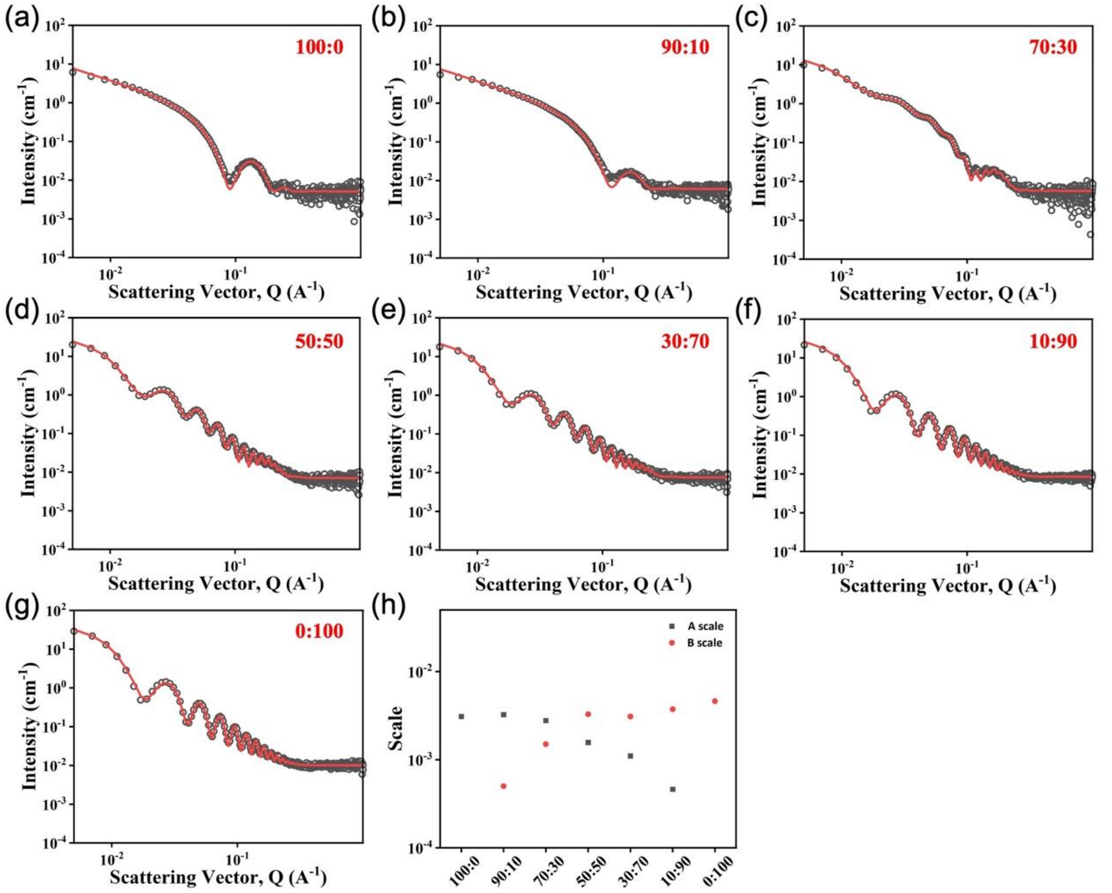

Figure S24. (a-g) SANS data (black circle) and fits (red line) for binary system solutions with volume ratios from 100:0 to 0:100 of 10 mg/mL (L,L)-, (L,D)-2NapFF stock solutions. (h) Plots showing scales of the two models obtained from fits against volume ratio.

Table S4. Summary of SANS data fitting parameters of binary system solutions with volume ratios from 100:0 to 0:100 of 10 mg/mL (L,L)-, (L,D)-2NapFF stock solutions.

	100:0	90:10	70:30	50:50	30:70	10:90	0:100
Scale		1	1	1	1	1	
Background (cm^{-1})	$5.22\times10^{-3}\pm7.58\times10^{-5}$	$5.97\times10^{-3}\pm7.10\times10^{-5}$	$5.60\times10^{-3}\pm8.589\times10^{-5}$	$7.44\times10^{-3}\pm8.45\times10^{-5}$	$7.83\times10^{-3}\pm7.50\times10^{-5}$	$8.69\times10^{-3}\pm8.75\times10^{-5}$	$1.02\times10^{-2}\pm5.76\times10^{-5}$
A Scale	$3.09\times10^{-3}\pm2.256\times10^{-5}$	$3.24\times10^{-3}\pm2.25\times10^{-5}$	$2.77\times10^{-3}\pm2.68\times10^{-5}$	$1.57\times10^{-3}\pm5.04\times10^{-5}$	$1.10\times10^{-3}\pm1.19\times10^{-4}$	$4.61\times10^{-3}\pm7.40\times10^{-4}$	
A Radius (Å)	16.5±0.1	8.7±0.1	10.3±0.2	13.4±0.3	15.3±0.6	42.4±3.8	
A Thickness (Å)	19.3±0.2	21.9±0.2	19.9±0.2	14.1±0.5	9.2±1.0	4.5±7.3	
A Length (Å)	2000	2000	2000	2000	2000	2000	
B Scale		$5.00\times10^{-4}\pm$	$1.50\times10^{-3}\pm$	$3.28\times10^{-3}\pm1.29\times10^{-5}$	$3.08\times10^{-3}\pm1.18\times10^{-5}$	$3.73\times10^{-3}\pm1.00\times10^{-5}$	$4.61\times10^{-3}\pm2.25\times10^{-5}$
B Radius (Å)		131.4	131.4	131.4	131.4	131.4	131.4±0.1
B Thickness (Å)		17	17	17	17	17	17.0±0.1
B Length (Å)		491	491	491	491	491	491.1±4.8
χ^2	2.09	3.03	2.91	4.93	5.71	9.80	8.00

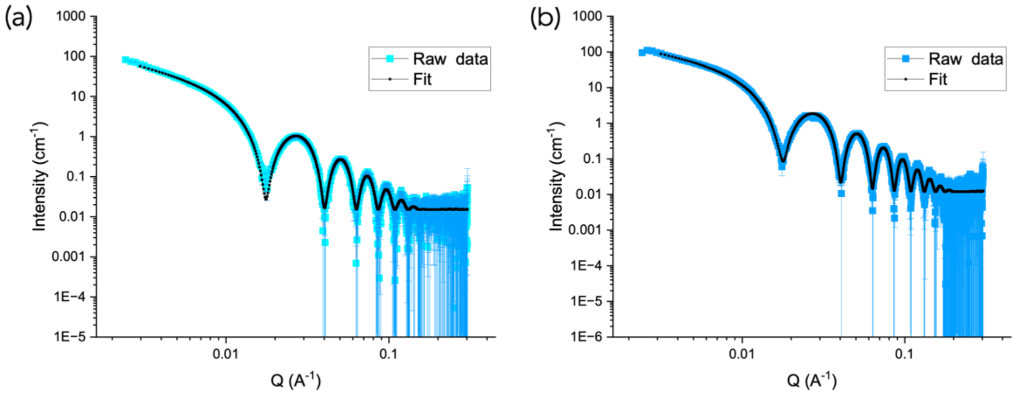

Figure S25. SAXS data (black circle) and fit (red line) for a solution of (L,D)-2NapFF alone at (a) a concentration of 5 mg/mL and (b) a concentration of 10 mg/mL. The fits are to a hollow cylinder model. For (a), the fit implies the radius is 12.0 nm and the thickness 3.5 nm. For (b), the fit implies the radius is 12.3 nm and the thickness 2.8 nm.

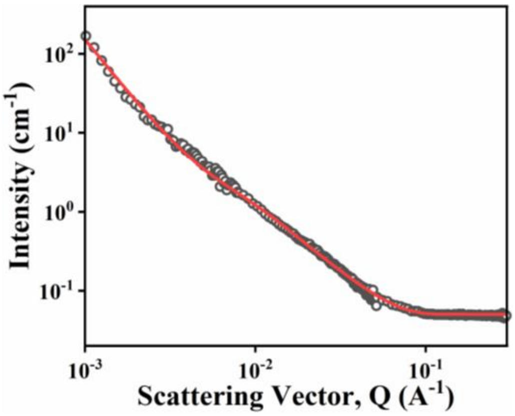

Figure S26. SANS data (black circle) and fit (red line) for a solution of (L,L)-2NapFF alone at a concentration of 2 mg/mL. The fit is to a flexible cylinder model combined with a power law, with a radius of 3.2 nm.

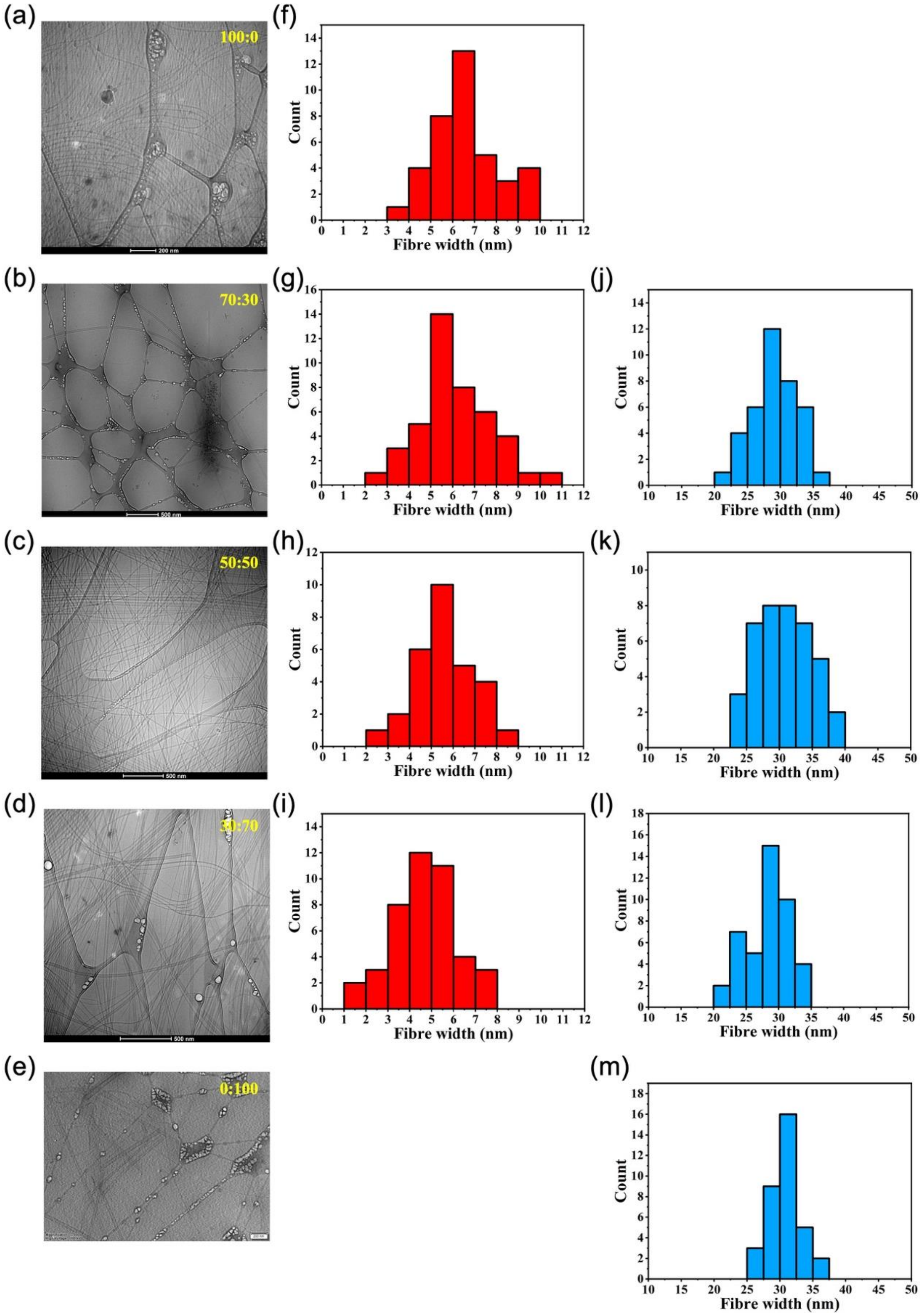

Figure S27. (a-e) Cryo-TEM image, histogram of width distribution of nanofibers formed by (f-i) (L,L)-2NapFF and (j-m) (L,D)-2NapFF component in binary system solutions with various volume ratios (100:0, 70:30, 50:50, 30:70 and 0:100) of 10 mg/mL (L,L)-, (L,D)-2NapFF stock solutions.

Table S5. Summary of average diameters of structures in binary system solutions with various volume ratios (100:0, 70:30, 50:50, 30:70 and 0:100) of 10 mg/mL (L,L)-, (L,D)-2NapFF stock solutions through analysis of corresponding cryo-TEM images.

Volume ratio	Diameter of (L,L)-2NapFF component	Diameter of (L,D)-2NapFF component
100:0	6.60±1.57	
70:30	6.12±1.70	29.14±3.41
50:50	5.67±1.35	30.61±3.95
30:70	4.78±1.37	28.40±3.28
0:100		30.87±2.53

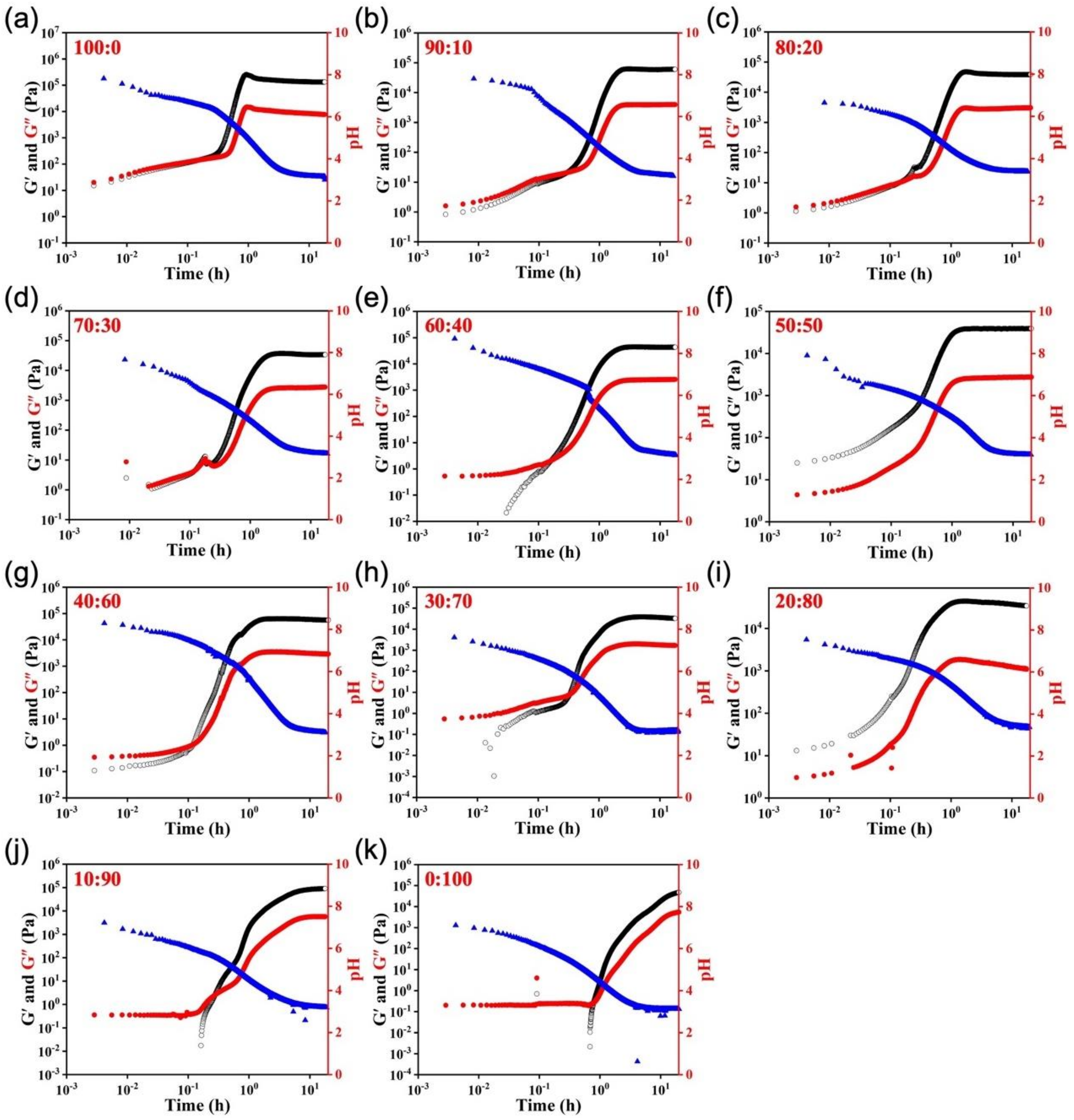

Figure S28. Time sweep (black circle and red dot) and pH monitoring (blue triangle) of binary system solutions with volume ratios from 100:0 to 0:100 of 10 mg/mL (L,L)-, (L,D)-2NapFF stock solutions after the addition of GdL.

Table S6. Summary of gelation time and pH of gel of binary systems with volume ratios from 100:0 to 0:100 of 10 mg/mL (L,L)-, (L,D)-2NapFF stock solutions after the addition of GdL.

Volume ratio	Gelation time (min)	pH of gel
100:0	9.3	3.15
90:10	17.3	3.15
80:20	9.0	3.38
70:30	7.3	3.16
60:40	9.7	3.15
50:50	0	3.19
40:60	7.35	3.10
30:70	19.8	3.19
20:80	0	3.38
10:90	16.6	3.19
0:100	47.0	3.12

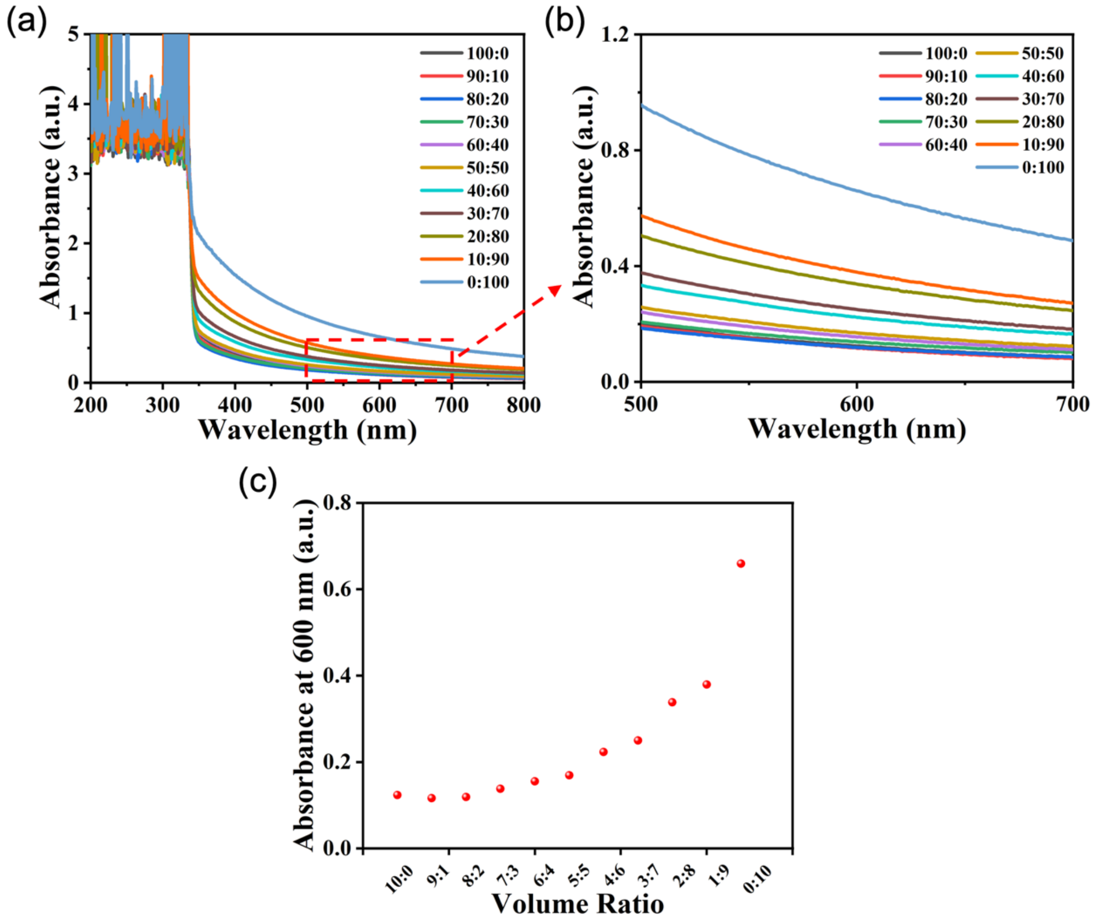

Figure S29. (a) UV-Vis absorption spectra of gels containing 10 mg/mL of (L,L)- and (L,D)-2NapFF at ratios ranging from 100:0 to 0:100 collected using a 2 mm path length quartz cuvette at 25 °C. (b) Spectra depicted the enlarged regions of the graph (a) at wavelengths of 500-700 nm. (c) Change in turbidity over composition at 600 nm for gels.

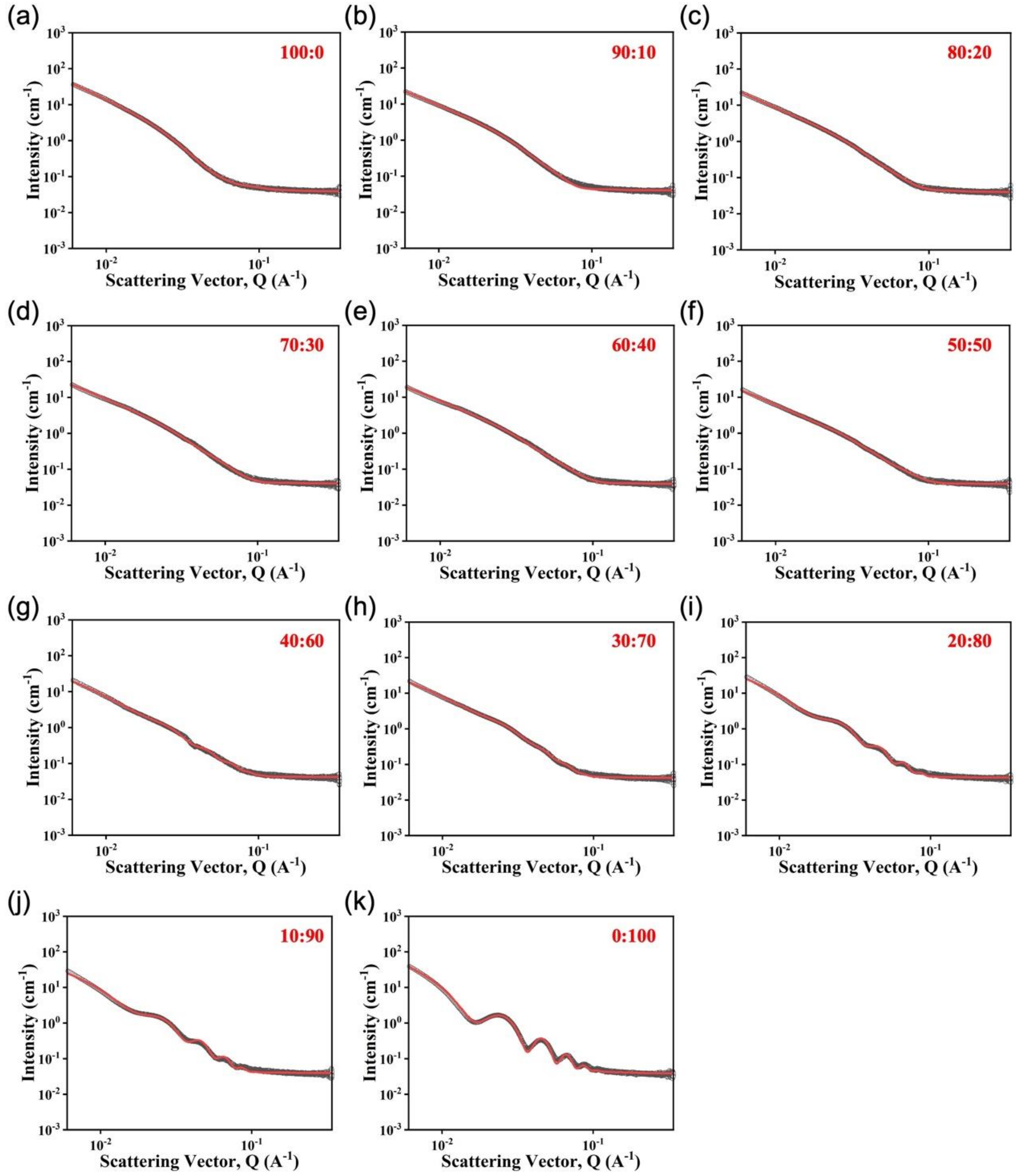

Figure S30. (a-k) SANS data (black circle) and fits (red line) for binary system gels with volume ratios from 100:0 to 0:100 of 10 mg/mL (L,L)-, (L,D)-2NapFF stock solutions.

Table S7. Summary of SANS data fitting parameters for the binary system gels with volume ratios from 100:0 to 0:100 of 10 mg/mL (L,L)-, (L,D)-2NapFF stock solutions. The abbreviations FEC, HC and PL represent flexible elliptical cylinder, hollow cylinder and power law, respectively.

	100:0	90:10	80:20	70:30	60:40	50:50	40:60	30:70	20:80	10:90	0:100
Model	FEC+PL	FEC	FEC+PL	FEC	FEC+PL	FEC+HC	FEC+HC	FEC+HC	FEC+HC	FEC+HC	HC+PL
scale	1		1		1	1	1	1	1	1	1
Background (cm^{-1})	$3.85\times10^{-2}\pm4.26\times10^{-5}$	$4.06\times10^{-2}\pm3.36\times10^{-5}$	4.05×10^{-2}	$4.03\times10^{-2}\pm3.49\times10^{-5}$	$3.95\times10^{-2}\pm3.29\times10^{-5}$	$3.89\times10^{-2}\pm3.56\times10^{-5}$	$4.19\times10^{-2}\pm3.94\times10^{-5}$	$4.20\times10^{-2}\pm3.70\times10^{-5}$	$4.27\times10^{-2}\pm3.77\times10^{-5}$	$3.95\times10^{-2}\pm3.47\times10^{-5}$	$3.84\times10^{-2}\pm3.43\times10^{-5}$
A_scale	$2.35\times10^{-4}\pm2.90\times10^{-6}$	$3.61\times10^{-4}\pm0.61\times10^{-6}$	$3.22\times10^{-4}\pm0.82\times10^{-6}$	$3.49\times10^{-4}\pm0.67\times10^{-6}$	$4.26\times10^{-4}\pm0.19\times10^{-6}$	$2.94\times10^{-4}\pm0.81\times10^{-6}$	$2.02\times10^{-4}\pm0.41\times10^{-6}$	$2.86\times10^{-1}\pm6.83\times10^{-4}$	$2.31\times10^{-4}\pm0.59\times10^{-6}$	$1.35\times10^{-4}\pm0.44\times10^{-6}$	
A_length (Å)	5000	5000	5000	5000	5000	5000	5000	5000	5000	5000	
A_kuhn_length (Å)	87.4±1.6	224.6±1.1	250.6±2.3	209.0±1.3	90.2	172.8±1.3	147.7±2.2	196.6±1.4	141.1±3.0	500.0±8.1	
A_radius (Å)	49.9±0.12	35.3±0.03	31.7±0.06	28.9±0.03	25.0	24.9±0.04	26.3±0.09	31.5±0.04	30.2±0.10	30.5±0.06	
A_axis_ratio	1.9±0.01	2.7±0.01	2.8±0.01	3.2±0.01	2.3	3.0±0.01	3.5±0.01	2.7±0.01	2.7±0.02	4.8±0.02	
B_scale						$0.22\times10^{-4}\pm5.21\times10^{-7}$	$0.95\times10^{-4}\pm6.16\times10^{-7}$	$6.51\times10^{-2}\pm5.61\times10^{-4}$	$2.86\times10^{-4}\pm6.35\times10^{-7}$	$3.38\times10^{-4}\pm6.48\times10^{-7}$	$6.36\times10^{-4}\pm7.87\times10^{-7}$
B_radius (Å)						124.8	124.8	124.8	124.8	124.8	$124.8\pm4.63\times10^{-2}$
B_thickness (Å)						51.452	51.452	51.452	51.452	51.452	$51.452\pm8.03\times10^{-2}$
B_length (Å)						5000	5000	5000	5000	5000	5000
scale	$2.43\times10^{-5}\pm3.51\times10^{-7}$		$1.11\times10^{-5}\pm4.40\times10^{-7}$		$2.79\times10^{-8}\pm2.81\times10^{-10}$						$1.39\times10^{-5}\pm9.28\times10^{-8}$
power	$2.5\pm4.26\times10^{-3}$		$2.6\pm7.11\times10^{-3}$		3.5						$2.7\pm1.54\times10^{-3}$
χ^2	1.31	5.77	1.87	8.39	9.67	2.85	6.27	3.96	11.00	19.02	22.15
